# A comprehensive survey of the mutagenic impact of common cancer cytotoxics

**DOI:** 10.1186/s13059-016-0963-7

**Published:** 2016-05-09

**Authors:** Bernadett Szikriszt, Ádám Póti, Orsolya Pipek, Marcin Krzystanek, Nnennaya Kanu, János Molnár, Dezső Ribli, Zoltán Szeltner, Gábor E. Tusnády, István Csabai, Zoltan Szallasi, Charles Swanton, Dávid Szüts

**Affiliations:** Institute of Enzymology, Research Centre for Natural Sciences, Hungarian Academy of Sciences, 1117 Budapest, Hungary; Department of Physics of Complex Systems, Eötvös Loránd University, 1117 Budapest, Hungary; Center for Biological Sequence Analysis, Department of Systems Biology, Technical University of Denmark, 2800 Lyngby, Denmark; CRUK Lung Cancer Centre of Excellence, UCL Cancer Institute, London, UK; Computational Health Informatics Program (CHIP), Boston Children’s Hospital, Boston, MA USA; Harvard Medical School, Boston, MA 02215 USA; Francis Crick Institute, 44 Lincoln’s Inn Fields, London, WCA2 3PX UK; MTA-SE-NAP, Brain Metastasis Research Group, 2nd Department of Pathology, Semmelweis University, 1091 Budapest, Hungary

**Keywords:** Whole genome sequencing, Mutagenesis, Cisplatin, Cyclophosphamide, Etoposide, Cytotoxics, Cancer chemotherapy, Chemotherapy resistance, BRCA2, Spontaneous mutagenesis, DT40

## Abstract

**Background:**

Genomic mutations caused by cytotoxic agents used in cancer chemotherapy may cause secondary malignancies as well as contribute to the evolution of treatment-resistant tumour cells. The stable diploid genome of the chicken DT40 lymphoblast cell line, an established DNA repair model system, is well suited to accurately assay genomic mutations.

**Results:**

We use whole genome sequencing of multiple DT40 clones to determine the mutagenic effect of eight common cytotoxics used for the treatment of millions of patients worldwide. We determine the spontaneous mutagenesis rate at 2.3 × 10^–10^ per base per cell division and find that cisplatin, cyclophosphamide and etoposide induce extra base substitutions with distinct spectra. After four cycles of exposure, cisplatin induces 0.8 mutations per Mb, equivalent to the median mutational burden in common leukaemias. Cisplatin-induced mutations, including short insertions and deletions, are mainly located at sites of putative intrastrand crosslinks. We find two of the newly defined cisplatin-specific mutation types as causes of the reversion of BRCA2 mutations in emerging cisplatin-resistant tumours or cell clones. Gemcitabine, 5-fluorouracil, hydroxyurea, doxorubicin and paclitaxel have no measurable mutagenic effect. The cisplatin-induced mutation spectrum shows good correlation with cancer mutation signatures attributed to smoking and other sources of guanine-directed base damage.

**Conclusion:**

This study provides support for the use of cell line mutagenesis assays to validate or predict the mutagenic effect of environmental and iatrogenic exposures. Our results suggest genetic reversion due to cisplatin-induced mutations as a distinct mechanism for developing resistance.

**Electronic supplementary material:**

The online version of this article (doi:10.1186/s13059-016-0963-7) contains supplementary material, which is available to authorized users.

## Background

Cytotoxic drugs have been in use for cancer therapy since the 1950s, and remain the first line treatment for most cancers today. These drugs inhibit cell proliferation through a range of different mechanisms, including directly damaging DNA, interfering with DNA metabolism and interfering with the mitotic machinery. Successful treatments kill tumour cells, but also exert side effects attributable to a number of factors including the inhibition of cell proliferation in healthy tissues. Treatments may also have long-term negative consequences through inducing genomic changes. In normal somatic cells, mutations induced by chemotherapy may accelerate tumorigenic processes. The development of secondary malignancies is an especially significant issue following childhood cancers and epidemiological studies have associated treatment with alkylating agents and topoisomerase inhibitors with the later development of acute myoblastic leukaemia (AML) and other tumour types [1]. Moreover, treatment-induced mutations in surviving cancer cells increase the genetic heterogeneity of the tumour and may contribute to the development of resistance to further treatment.

Chemotherapeutics are tested for genotoxicity, the ability of the drug to cause DNA damage. The most important currently approved tests are the comet assay for detecting DNA breaks, the chromosome aberration assay and the micronucleus formation test [2]. These assays give indirect and imprecise predictions of carcinogenic potential [3], as a finding of genotoxicity only reveals that a compound has potential to cause genomic mutations, without measuring the outcome in a surviving cell. Mutagenicity itself has primarily been assayed using reporter genes, including the Ames reverse mutation assay in bacteria [4] and *HPRT* mutagenesis in mammalian cell lines [5]. However, the comprehensive detection of all genomic changes of all types only became available with affordable whole genome sequencing.

Mutagenic effects have been attributed to a large proportion of cancer chemotherapeutic agents. Alkylating agents induce direct DNA adducts and nitrogen mustards such as cyclophosphamide have been shown to induce base substitution mutations in mutation reporters as well as chromosome rearrangements [6]. Platinum-containing crosslinking agents work by a similar mechanism to alkylating agents. Cisplatin adducts have been shown to cause base substitutions in vitro and in reporter genes [7], which were also detected in cisplatin-treated *C. elegans* worm genomes [8]. Topoisomerase II inhibitors such as etoposide and doxorubicin cause DNA breaks, which are the likely causes of chromosomal translocations in secondary cancers induced by these drugs [9, 10]. Drugs of the diverse antimetabolite family interfere with DNA replication, leading to double strand breaks and chromosome aberrations [11–13]. The microtubule-targeted class of cancer chemotherapeutics are not expected to have a direct impact on mutagenesis, though paclitaxel has been described to affect DNA repair through disrupting the trafficking of DNA repair proteins [14].

In summary, while genotoxic effects have been measured indirectly for most cytotoxic drugs, sequence-based data for mutagenicity are only available for cisplatin, from an invertebrate model [8]. To acquire reliable data on genomic mutagenicity, we performed whole genome sequencing on cultured cells treated with representatives of each major category of cancer chemotherapeutics. Each of the chosen cytotoxic agents (Table 1) has been reported to give a positive result in the Ames test or the related bacterial umu-test [15–19]. *HPRT* mutagenesis was reported for cisplatin, cyclophosphamide, doxorubicin and etoposide [20–23], but absent for hydroxyurea [24]. We set out to determine how relevant these findings are to genomic mutagenesis in vertebrate cells. Such studies have not been performed previously, but a proof-of-concept is provided by a recent report on the genomic effect of three environmental mutagens in single sequenced mouse embryonic fibroblast clones [25] as well as earlier studies that used whole exome sequencing [26–28]. The main benefit of the obtained mutagenic spectrum data will be the ability to use cancer genome sequences to determine whether the mutagenic drugs have contributed to the development of the tumour, and we provide an important example for this in the reversion of oncogenic gene mutations. The chicken DT40 lymphoblastoma cell line was chosen for treatments for the following reasons: (1) the genome size is about one-third compared to the human genome; (2) this cell line has been used very extensively for DNA repair studies and it models mammalian DNA repair well [29]; and (3) the availability of a wide range of isogenic DNA repair mutant cell lines will allow future comparisons on the influence of individual repair factors on mutagenesis. This detailed genomic analysis of multiple post-treatment cell clones provides the most comprehensive survey of the mutagenic potential of commonly used cytotoxics in cancer medicine.

**Table 1:** Cytotoxic drugs investigated in this study

Drug	Class	Mechanism	DT40 treatment duration	DT40 treatment concentration	DT40 IC_50_	Clinical usage	Clinical usage
						Total plasma concentration	Reference
Cisplatin	Alkylating-like agent	DNA adducts, crosslinks	1 h	10 μM	9.4 μM	1.3–3.9 μM	[70]
Cyclophosphamide	Alkylating-like agent	DNA adducts, crosslinks	1 h	30 mM	67 mM	38.3–76.6 μM	[71]
Hydroxyurea	Antimetabolite	Ribonucleotide reductase inhibition	24 h	20 μM	22 μM	150 μM–1 mM	[72]
Gemcitabine	Antimetabolite	Nucleoside analogue	24 h	6 nM	10.9 nM	53.2 μM	[73]
5-Fluorouracil	Antimetabolite	Nucleoside analogue, thymidylate synthase inhibition	24 h	6 μM	13.3 μM	770 nM–5.4 μM	[74]
Etoposide	Topoisomerase inhibitor	Topoisomerase II inhibition	24 h	200 nM	234 nM	46–194 nM	[75]
Doxorubicin	Anthracycline	DNA intercalation, topoisomerase II inhibition	24 h	2 nM	1.69 nM	73.6 nM–1.16 μM	[76]
Paclitaxel	Anti-microtubule agent	Stabilises microtubules, blocks mitosis	24 h	40 nM	34 nM	1.5–6 μM	[77]

The name, class and basic mechanism of each drug used in this study is shown, together with the duration and concentration of mutagenesis assay treatments, the estimated IC_50_ concentrations under the same treatment conditions and data on the total plasma concentration range reported in clinical use, with the matching literature reference

## Results

### In vitro use of eight chemotherapeutic agents

**Fig. 1:**
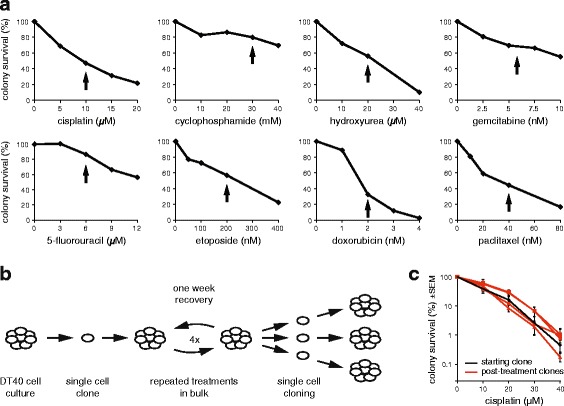
Cytotoxic treatments. **a** Colony survival assay of DT40 cells treated with the indicated cytotoxic drugs for 1 h (cisplatin, cyclophosphamide) or 24 h. The concentration chosen for mutagenesis assays are indicated with *black arrows*. **b** A *schematic drawing* of the mutagenesis assay. Genomic DNA was sequenced from the pre-treatment starting cell clone and three post-treatment cell clones. **c** Comparison of the cisplatin sensitivity of the starting clone (*black*) and clones isolated following four rounds of cisplatin treatment (*red*). Mean and SEM of three measurements is shown

Isogenic wild-type DT40 cells derived from a single cell clone were treated with eight different commonly used cytotoxic agents representing each of the main classes of cancer chemotherapeutics. The agents are listed in Table 1. To select a treatment concentration, we measured the sensitivity of DT40 cells to each drug using a clonogenic survival assay (Fig. 1a). We chose treatment conditions near the IC_50_ concentration of each drug that induce only moderate cell death, with 30–85 % of the cells surviving, in order to avoid selecting for resistant clones that could behave differently during subsequent treatment rounds due to potential changes in, for example, drug transport or DNA repair. For the surviving cells, treatments were repeated once a week through four cycles, mimicking cancer chemotherapy regimens and increasing the chance of inducing mutations (Fig. 1b). A comparison of the cisplatin sensitivity of several post-treatment clones to the starting clone shows that this moderate treatment regimen did not cause significant selection for resistance (Fig. 1c).

One of the tested drugs, cyclophosphamide, undergoes activation by hydroxylation by cytochrome P450 enzymes [30]. While this is thought to mainly take place in the liver during therapeutic treatment, lymphocytes have also been shown to express the enzymes necessary for cyclophosphamide activation [31, 32]. Therefore, due to the instability and limited availability of the active metabolite 4-hydroxycyclophosphamide, cyclophosphamide was added to cells in its pro-drug form. Cisplatin and cyclophosphamide, the two drugs that are known to form DNA adducts, were added for 1 h with the reasoning that their DNA damaging effect should be largely independent of cell cycle phase. The remaining drugs were used in 24-h treatments. This duration is twice the length of the DT40 cell cycle, ensuring that each cell would be affected by the treatment regardless of the cell cycle phase in which the drugs exert their main effect.

Single nucleotide variation (SNV) and short insertion/deletion mutations were identified in three cell clones derived from each treatment using the IsoMut method developed for this purpose [33, 34]. This approach provides mutation information for the genomes of three individual cells that went through the treatment regime (Fig. 1b). Briefly, we compared all the whole genome sequences obtained in this study at each genomic position, and only accepted a mutation if it was present in exactly one sample, satisfying criteria on minimum mutated allele frequency, coverage of the mutated sample, and minimum reference allele frequency of each other sample. Due to the lack of availability of validated SNP and short insertion or deletion mutations (indel) datasets, this mutation detection method performs much better on the chicken genome than other commonly used methods, identifying 90–95 % of all mutations with no more than 0–5 false-positive SNVs per genome [33].

### Spontaneous mutations

**Fig. 2:**
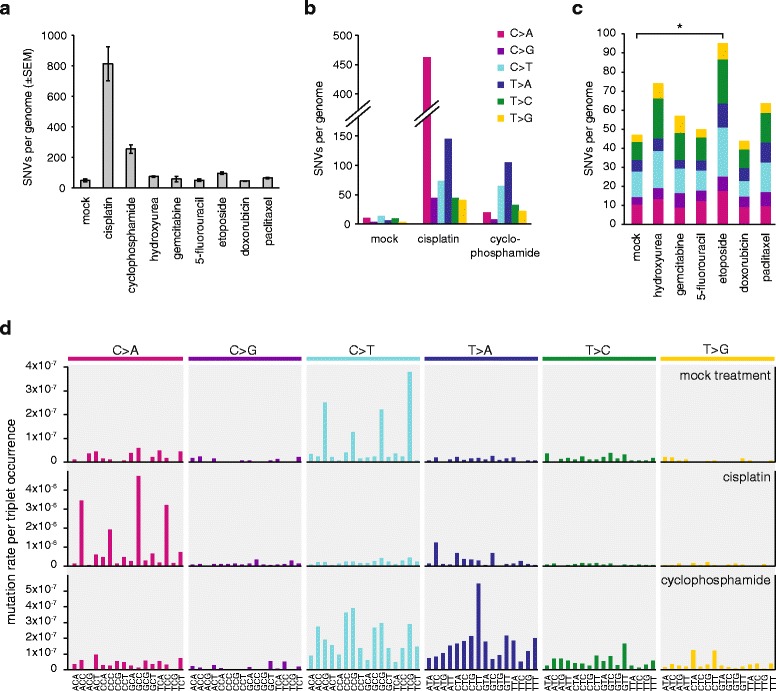
Number and spectrum of treatment-induced SNVs. **a** The mean number of observed SNVs per genome following the described treatment regimen with the indicated drugs. *Error bars* indicate SEM. **b** Base substitution spectrum of mutations that arose from the mock treatment, as well as cisplatin and cyclophosphamide treatments. **c** The mean number of mutations per sample and base substitution spectrum of the indicated treatments. Significant differences from the mock treatment (*p* <0.05, Student’s t-test) are indicated with an *asterisk*. **d** Triplet mutation spectra of the mock, cisplatin and cyclophosphamide treatments. The middle base of each triplet, listed at the *bottom*, mutated as indicated at the *top of the panel*. The number of mutations of each type was normalised to the frequency of occurrence of that base triplet in the chicken genome, and the resulting mutation rates are shown

Following a mock treatment regimen spanning approximately 100 cell generations, we detected 47 ± 20 (SD) novel SNVs in three post-treatment clones (Table 2, Additional file 1: Table S1). It is likely that almost all the identified mutations truly arose during the mock treatment, as these were identified as unique mutations among all the whole genome sequences obtained for this study, and the same mutation detection method found no unique SNVs – which would be false positives – in the pre-treatment starting clone (Table 2). Of the six possible base substitutions (C > A, C > G, C > T, T > A, T > C, T > G), C > T transitions and C > A transversions were the most common among the spontaneous mutations (Fig. 2c, d). The observed mutation number, projected to the 2.06 × 10^9^ base pair diploid genome is equivalent to about 2.3 × 10^–10^ mutations per base per cell division. When mutations are viewed in the context of the neighbouring bases, and the spontaneous ‘triplet mutation spectrum’ is normalised to the frequency of genomic occurrence of each triplet, it becomes apparent that NCG > NTG mutations are most common, presumably due to C > T base substitutions at methylated CpG sequences [35]. We calculated that NCG > NTG mutations were 15× more common than the mean mutation rate. Non-normalised triplet spectra are shown in Additional file 2: Figure S1.

**Table 2:** Number of SNV and short insertion/deletion mutations in the sequenced samples

Treatment	n	SNV	Insertion	Deletion
		Mean ± SD	Mean ± SD	Mean ± SD
None (starting clone)	1	0	0	0
Mock	4	47 ± 20	4.5 ± 1.3	3.0 ± 0.8
Cisplatin	3	812 ± 193	49.0 ± 15.0	83.0 ± 21.2
Cyclophosphamide	3	254 ± 50	3.0 ± 1.7	5.0 ± 1.7
Hydroxyurea	3	74 ± 9	4.7 ± 1.5	3.7 ± 1.2
Gemcitabine	3	57 ± 31	2.3 ± 2.1	3.0 ± 1.7
5-fluorouracil	3	50 ± 16	3.0 ± 1.0	2.7 ± 1.5
Etoposide	3	95 ± 15	3.7 ± 1.2	6.7 ± 4.2
Doxorubicin	3	44 ± 15	3.3 ± 0.6	4.0 ± 4.6
Paclitaxel	3	64 ± 10	1.0 ± 1.0	3.0 ± 0.0

### Cisplatin induces base substitutions and short indels

**Fig. 3:**
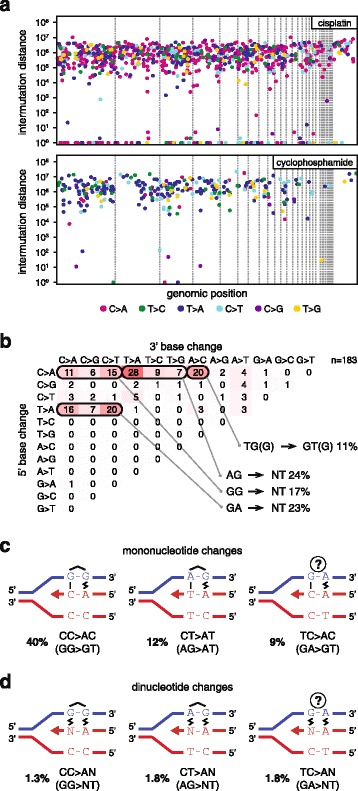
SNV mutation spacing, dinucleotide mutations and proposed mechanisms of mono- and dinucleotide mutations. **a** The distance of each SNV mutation from the previous SNV on the same chromosome is plotted against the genomic position of the mutation. *Thin dashed lines* indicate chromosome boundaries. Chromosomes are shown in numerical order; chromosome Z is shown last on the *right*. The colour of each *dot* illustrates the type of mutation according to the key at the bottom of the panel. Mutations with an intermutation distance of one are part of dinucleotide mutations. One sequenced clone of each is shown. **b** *Sequence analysis* of the 183 dinucleotide mutations detected following cisplatin treatment. The change in the 5’ base is shown in the rows, while the 3’ base in the columns. The equivalent mutations on the two strands are added together, e.g. GG > TT is shown as CC > AA. The most common mutation types are grouped together below the table and their sequences are indicated using the purine-rich strand to aid interpretation. **c** *Schematic models* for the replicative process that may generate each of the most common classes of cisplatin-induced mononucleotide (**c**) and dinucleotide (**d**) mutations. Putative intrastrand crosslinks are marked, the uncertain lesion at mutated GA sequences is indicated with a *question mark*. Non-canonical base pairing is shown with a *zig-zag symbol*. The contribution of each mutation class to the total number of observed SNVs is shown

Cisplatin induced the greatest number of SNVs among the eight tested drugs (Fig. 2a). We performed a detailed analysis of cisplatin-induced mutations to better understand the mutagenic mechanisms. We detected 812 ± 193 SNVs per sequenced post-treatment clone. C/G > A/T transversions were most common, accounting for 57 % of all SNVs, but all six classes of base substitutions increased at least fourfold (Fig. 2b). Looking at cisplatin-induced SNVs in the context of the neighbouring bases, it is apparent that NCC > NAC mutations are most common, accounting for 40 % of all SNV cases. Further common changes are NCT > NAT and NTC > NAC, arising in 12 % and 9 % of the SNV cases (Fig. 2d, Additional file 2: Figure S1 and Figure S3 and Additional file 1: Table S2). As the overwhelming majority of cisplatin-induced DNA lesions are intrastrand crosslinks between neighbouring purines [36, 37], these three SNV types could represent mutations opposite the 3’ base of crosslinked GG, AG and GA dinucleotides, respectively. In case of GG and AG intrastrand crosslinks, these mutations arise through the incorrect incorporation of an adenosine opposite the 3’ G of the lesion (Fig. 3c). However, GA crosslinks have not been observed in the above reports. Therefore, we catalogued the bases surrounding the 211 observed TC > AC (GA > GT) mutations, and found that 159 incidences happened at TCC > ACC or TCT > ACT sequences, suggesting that the adjacent base pair 3’ to a GG or AG intrastrand crosslink can also mutate. Of the remaining 52 mutations, ten happened at the 5’ base of potential AG crosslinks at CTC > CAC sequences, but in the remaining cases the only potential site for a bipurine crosslink is at GA (Additional file 2: Figure S2). We conclude that cisplatin induces mutagenic lesions at GA dinucleotides, where the lesions may be hitherto unobserved intrastrand GA crosslinks or monoadducts. To complete the analysis of cisplatin-induced single nucleotide mutations, we note an enrichment of CCA > CAA and CTN > CAN base changes, suggesting adenosine mis-incorporation opposite the 5’ base of crosslinked GG or AG dinucleotides.

In agreement with finding mutations (pyrimidine to adenine) across both 3’ and 5’ bases of putative crosslinked intrastrand cisplatin lesions, we also detected 61 ± 21 dinucleotide mutations per sample (Fig. 3a, Additional file 1: Table S3). Seventy-five percent of these mutations were found at AG, GG or GA dinucleotides. Interestingly, these changed to a range of sequences, equivalent to the incorporation of dinucleotides AA, AT, AC and AG opposite the putative intrastrand crosslink (Fig. 3d). A further common dinucleotide mutation class was CA > AC and 18 of 20 cases were found at CCA sequences. On the opposite strand these TGG > GTG mutations could indicate base changes in the position 5’ to GG crosslinks. The classification of different dinucleotide mutations is shown in Fig. 3b.

Taken together, we observed base substitution mutations at the 5’ position and the 3’ position, as well as the preceding and the following position of putative intrastrand crosslinks. Sequencing the replicated outcome of a GG crosslink in a shuttle plasmid only provided sufficient evidence of mutations at the 3’ position [38] and the number of mutations detected in cisplatin-treated *C. elegans* worms allowed the detection of the same mutations, as well as dinucleotide mutations at probable AG crosslinks [8]. Our high resolution data indicate mutagenesis at each position of a 4-base pair stretch centred on crosslinked GG and AG lesions.

Cisplatin-induced interstrand crosslinks form at GC sequences [39], which can only be present in the triplet spectrum data as GCN. Assuming that by analogy with mutations seen at putative intrastrand crosslinks the most common cause of mutations at interstrand crosslink lesions would be adenosine misincorporation opposite central crosslinked G, these mutations should present as GCN > GAN changes. Some of these triplet base changes were already counted above as potential intrastrand crosslink-induced mutations. Only the GCG > GAG combination could not happen at the site of an intrastrand crosslink, as GCG contains no neighbouring purines. These mutations are very rare (0.2 % of all SNVs) after cisplatin treatment. In conclusion, our data do not show strong evidence of point mutations induced by cisplatin interstrand crosslink adducts.

**Fig. 4:**
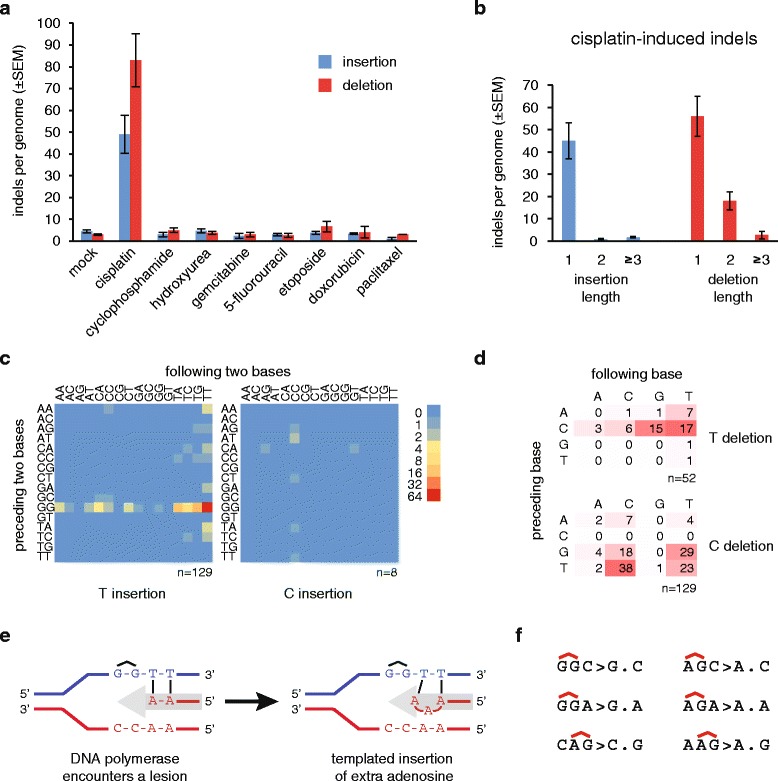
Cisplatin-induced insertions and deletions. **a** The mean number of observed insertions (*blue*) and deletions (*red*) per genome following the described treatment regimen with the indicated drugs. *Error bars* indicate SEM. **b** Length distribution of cisplatin-induced insertions and deletions. **c** *Heat map* of the frequency of one-base insertions, classified according to the preceding and the following two bases as indicated. The inserted base is shown below each panel. The equivalent mutations on the two strands are added and shown as T or C insertions. **d** *Table* and *heat map* of the frequency of one-base deletions, classified according to the preceding and the following base as indicated. The equivalent mutations on the two strands are added and shown as T or C deletions, shown to the *right*. **e** A *schematic model* of the generation of the most common GGTT > GGTTT insertions during DNA replication. The incoming DNA polymerase (*grey arrow*) inserts adenosines opposite the thymine bases, then it inserts an extra adenosine upon encountering the cisplatin-induced GG intrastrand crosslink. **f** Sequence context of the most common one-base deletions shown on the purine rich strand, with the position of putative intrastrand crosslinks indicated above the sequence

Cisplatin treatment also induced a remarkable number of short insertion and deletion mutations, totalling 132 ± 34 per sample (Fig. 4a, Table 2, Additional file 1: Table S1). The insertions were almost exclusively one base long (95 % of all insertions, Fig. 4b). We classified one-base insertions based on their sequence context (Fig. 4c, Additional file 1: Table S4). Ninety-four percent of one-base insertions were A/T base pairs. On the strand with the thymidine insertion, the preceding two bases were GG in 81 % of cases, presumably representing the site of an intrastrand crosslink Surprisingly, the bases following the insertion site also showed strong sequence preference. The first base following a thymidine insertion was 84 % T, while the first two bases together were 51 % TT. If the mutagenic process is DNA synthesis using the damaged strand as template, we can conclude that it preferentially inserts an extra adenosine when the bases 3’ to the template GG crosslink are thymines (see Fig. 4e for a model). Six of the eight observed C/G base pair insertions occurred at CC/GG sites (Fig. 4c), also likely sites of intrastrand crosslinks.

Seventy-three percent of cisplatin-induced deletions were one base pair long. A classification of one base pair deletion based on the deleted base and the neighbouring two bases shows that the most common deletions affected GG or AG sequence motifs, which may be sites of intrastrand crosslinks (Fig. 4d, f, Additional file 1: Table S4). In the case of AG, based on the AGC > AC and CAG > CG deletions, it is possible to conclude that either the 3’ or the 5’ base pair of a putative crosslinked AG dinucleotide may get deleted. In agreement with this, 40 of 59 (68 %) observed two-base deletions removed both base pairs of putative AG or GG intrastrand crosslinks (Additional file 2: Figure S4).

### Cyclophosphamide primarily causes T > A and C > T mutations

Cyclophosphamide induced 254 ± 50 base substitution mutations, which is more than five times higher than the mock treatment (*p* = 0.025, Student’s t-test). The most common base changes were T > A and C > T, followed by a more modest increase in the number of T > C and T > G mutations (Fig. 2b). Cyclophosphamide has been shown to induce a range of adducts in the following proportions: N7-guanine monoadducts (22 %), crosslinked adducts (6–12 %) and phosphotriester adducts (67 %) [6]. The N7-guanine monoadducts or the G-G interstrand crosslinks may account for the C > T lesions. To look for evidence of crosslinked adducts, of which the most common have been observed as interstrand adducts between guanines at GNC sequences, we looked for sequence preferences two bases upstream from mutated cytosines (Additional file 2: Figure S3), but we could not find strong evidence for such changes. The prevalent T > A mutations, and also the rarer T > C and T > G mutations, preferentially occur at the centre of NTT triplets, with some further preference for CTT and TTT (Fig. 2d). These are unlikely to be caused by guanine adducts and may be due to phosphotriester adducts instead. Intermutation distances indicated few dinucleotide mutations or other clustering of mutations (Fig. 3a). There was no clustering of mutations when comparing different treated clones in case of either cisplatin or cyclophosphamide, suggesting the lack of mutational hotspots and the largely random distribution of SNVs (Additional file 2: Figure S5).

In contrast to cisplatin, cyclophosphamide treatment did not cause a significant increase in the number of insertion or deletion mutations (Fig. 4a).

### Etoposide treatment elevates the base substitution frequency

Six further drugs were investigated for their mutagenic potential: the antimetabolites hydroxyurea, gemcitabine and 5-fluorouracil, plus the topoisomerase II inhibitor etoposide, the anthracycline doxorubicin and the anti-microtubule agent paclitaxel. A comparison of the total SNV numbers for the mock treatment plus these six treatments by ANOVA revealed a significant difference (*p* = 0.025). Indeed, etoposide induced more than twice the number of mutations as the mock treatment, which is a significant pairwise difference (*p* = 0.017, Student’s t-test). Each base substitution category increased, resulting in no major change in the overall mutation spectrum (Fig. 2c). In contrast to SNVs, the number of indels was not significantly elevated compared to the mock treatment (Fig. 4a).

### No detectable mutagenic effect of hydroxyurea, gemcitabine, 5-fluorouracil, doxorubicin and paclitaxel

Twenty-four-hour treatments with hydroxyurea, gemcitabine, 5-fluorouracil, doxorubicin and paclitaxel did not induce a significant number of extra SNVs or indels in comparison to the mock treatment (Figs. 2a, 4a, ANOVA analysis). These treatments also did not change the spontaneous SNV mutation spectrum (Fig. 2c). Also, none of the tested agents, including cisplatin, cyclophosphamide and etoposide, induced any larger indels (over 100 bp) or genome rearrangement events, except for a 3453-bp deletion in one etoposide-treated clone.

In conclusion, at concentrations that kill a moderate proportion of cultured cells, none of hydroxyurea, gemcitabine, 5-fluorouracil, doxorubicin or paclitaxel induced measurable genomic mutagenesis. In contrast, in the same assay, cisplatin and cyclophosphamide induced a large number of mutations with distinct mutation spectra, while etoposide treatment resulted in marginally elevated base substitution mutagenesis.

### Lower mutagenesis rates in genes provides evidence of distinct repair rates

**Fig. 5:**
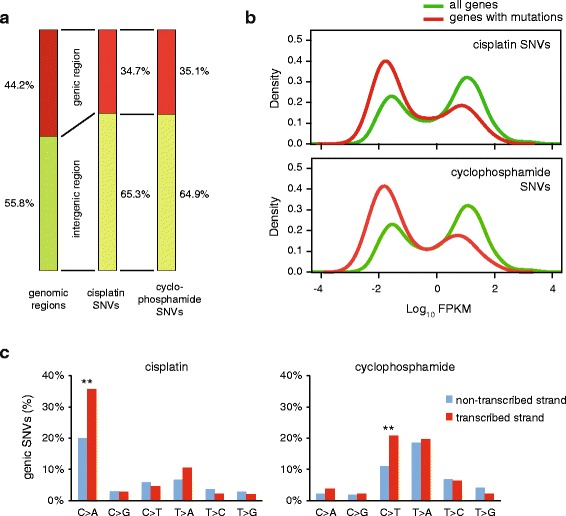
Mutation density with respect to gene transcription. **a** The proportion of genomic regions classified as intergenic (*green*) or genic (*red*) based on Ensembl genome annotation, shown on the *left*, differs from the proportion of cisplatin or cyclophosphamide-induced SNVs found in the respective regions (*middle and right columns*). **b** The distribution of the expression level of all genes, based on RNA-Seq coverage data (*green*) is shown against the distribution of the expression level of genes containing cisplatin or cyclophosphamide-induced SNVs (*red*). Expression levels are shown as the log_10_ of FPKM values (fragments per kilobase of transcript per million of mapped reads). **c** Strand bias of genic mutations induced by cisplatin (*left*) or cyclophosphamide (*right*). Highly significant (*p* <0.001, χ^2^ test) differences between the non-transcribed and the transcribed strands are indicated with *double asterisks*

Chemotherapy-induced mutations have the potential of altering gene function and thereby contributing to the development of resistance or secondary tumours. To gauge the importance of this effect, we mapped cisplatin- and cyclophosphamide-induced SNVs with respect to gene sequences. A total of 44.2 % of the chicken genome is annotated to code for primary transcripts in genome version Galgal4.82. Interestingly, a smaller proportion of treatment-induced mutations appeared at genes (34.7 % and 35.1 %) than expected from a uniform distribution (Fig. 5a). Relative to a uniform genomic distribution, mutations are 17 % more likely to occur at intergenic regions, while they are 22 % underrepresented at genic regions. The most likely explanation for the highly significant reduction of SNV numbers at genes versus intergenic regions (*p* <0.001 in case of both cisplatin and cyclophosphamide, χ^2^ test) is the activity of transcription-coupled repair (TCR), which can remove single strand lesions in an error-free manner [40]. We made use of an RNA-seq dataset from the DT40 cell line to ask whether the gene expression level influences mutation density, as expected if it is influenced by TCR. Indeed, we found that the distribution of mutated genes is skewed towards low expression (Fig. 5b), suggesting that the error-free repair of lesions is more efficient in highly expressed genes. Moreover, highly significant (*p* <0.001, χ^2^ test) strand bias of cisplatin-induced C > A and cyclophosphamide-induced C > T mutations in genes specifically points to efficient repair of guanine adducts in the transcribed strand by TCR (Fig. 5c).

### Correlation of the identified mutational patterns with mutational signatures in human cancer

**Fig. 6:**
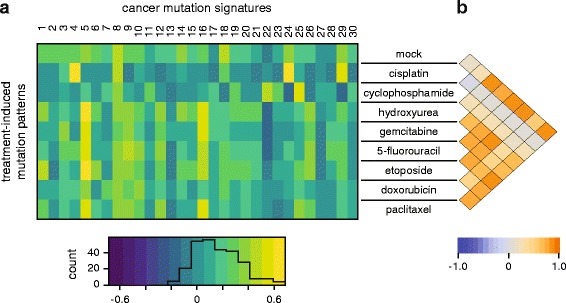
Correlation of drug-induced mutation patterns with mutation signatures identified in cancer. **a** *Heat map* of the Pearson correlation coefficient between triplet base mutation patterns induced by the cytotoxic treatment adjusted to human triplet frequencies (*rows*) and the 30 confirmed mutational signatures identified in human cancer [43]. The *heat map key* is shown at the *bottom*, with an overlaid histogram indicating the number of cells in each value range. **b** *Heat map* showing Pearson correlation coefficients between each pair of treatment-induced mutational patterns

We compared the various treatment-induced mutational patterns to mutational signatures identified in human cancer (COSMIC signatures) [41–43]. The normalised triplet spectrum of the mock treatment showed good visual similarity with the ageing-associated signature 1 (Fig. 1d) due to the presence of CG > TG mutations. However, this was not borne out in Pearson correlation analysis (Fig. 6a) as in our mock treated samples there is a range of mutation types in addition to the 15-fold overrepresented CG > TG mutations, while signature 1 essentially contains no other mutation types. The broad-spectrum signature 5 is also associated with ageing [42] and we observed positive correlation between this signature and several treatments that did not change the spontaneous mutation profile (Fig. 6a). In agreement with the dominance of ageing-related mutational processes, the mutation profiles of all treatments except cisplatin and cyclophosphamide show good pairwise correlation (Fig. 6b). Cisplatin-induced mutations correlate well with the smoking-specific signature 4 and the aflatoxin-induced signature 24 (Fig. 6a), suggesting that these agents cause mutations by similar mechanisms. Indeed, the three genotoxins all form bulky adducts at N7-guanines, which are generated by polycyclic aromatic hydrocarbons in the case of cigarette smoke [44]. Finally, cyclophosphamide-induced mutations show only weak correlation with the rare signature 25 of unknown aetiology (Fig. 6a). These results demonstrate that while mutagenesis analysis in cell lines can model the mutational processes observed in cancer, as also evidenced by exome sequencing of mutagen-treated mouse and human cells [26–28] and whole genome sequencing of individual mouse embryonic fibroblast clones [25], it is unlikely that mutations induced by cisplatin or cyclophosphamide treatment significantly contributed to COSMIC signatures.

### Mutagenic chemotherapy may induce resistance through genetic reversal of mutated genes

Mutagenic chemotherapy may have very significant consequences if the induced mutations contribute to the subclonal evolution of treatment resistance in surviving cells. We looked for evidence for such a process among documented mutations that restore functionality to mutated *BRCA1* or *BRCA2* genes [45]. Among seven frameshift mutations observed to restore *BRCA2* gene function and cause resistance following cisplatin treatment of Capan-1 cells that carry a single base deletion in *BRCA2* [46], we found two instances of GGT > GGTT insertions, which is by far the most common sequence of cisplatin-induced insertions (Fig. 4c). These insertions, 18 bp downstream of the deleted base pair, restored the reading frame, the protein level and the function of BRCA2 [46]. In a separate study, a nonsense mutation in *BRCA2* became inactivated by a TAG > TAT SNV in a cisplatin-treated ovarian adenocarcinoma following a cisplatin-resistant relapse. In the PEO1 cell line established from the tumour before the emergence of cisplatin resistance, cisplatin selection led to TAG > TTG mutations of the stop codon in eight out of eight resistant clones that restored the BRCA2 protein [47]. Our results show that mutation of either base to thymine at AG putative intrastrand crosslinks are a common consequence of cisplatin treatment, with CT > AT (AG > AT) and CT > CA (AG > TG) mutations making up 12 % and 7 % of all cisplatin-induced SNVs, respectively (Fig. 2d). The finding of these newly identified cisplatin-induced insertions and SNVs in BRCA2 revertants emerging in vivo and in vitro very strongly suggests that therapy can directly induce resistance-causing mutations.

We attempted to calculate an estimate for the likelihood of drug-induced mutations generating the exact genetic changes required to revert frameshift or nonsense mutations. If a reverting frameshift needs to happen within 20 base pairs of the original mutation and an SNV needs to change any one of the three bases of a stop codon, then 10^8^ randomly placed indels, or 7 × 10^8^ SNVs, would be required for a 50 % chance of the required mutation to occur in the human genome. Our experimental four-cycle cisplatin treatment regimen induced about 130 indels and 800 SNVs per Gb. If a clinical treatment regimen had the same mutagenic effect, it would take fewer than 1 million surviving treated tumour cells for a 50 % chance of the treatment-induced reversion of a frameshift or deletion of a nonsense mutation. Though partly based on speculative numbers, our approximations suggest that the effect of mutagenic treatments likely contributes to the evolution of drug resistance through the initiation of de novo mutations and indels in cancer subclones.

## Discussion

We have determined in unbiased whole genome analyses the mutagenicity of eight different common chemotherapeutics. Cisplatin was found to induce many base substitution mutations as well as very short insertion/deletion mutations, and the sequence context of these mutations suggests that they primarily arose at the site of intrastrand crosslinks. Cyclophosphamide also induces base substitution mutations with a specific spectrum, while six further drugs have little mutagenic effect, with a slight elevation of base substitutions after etoposide treatment. Our findings may be relevant to assessing the long-term outcome of treatment with the investigated cytotoxic drugs.

Mutagenesis assays are essential to test the mutation-causing effect of chemical agents that humans are exposed to, be they medications or environmental agents. In this study we used the genome of a vertebrate cell line for the purpose of a mutagenesis assay. Whole genome sequencing in the DT40 cell line far surpasses the currently used mutagenesis tests in its relevance to human biology: the commonly used bacterial Ames test takes place in a different metabolic and DNA repair environment, while reporter gene based tests in mammalian cells are affected by sequence bias due to the requirement that detected mutations must affect protein-coding sequences. We believe that cell line whole genome sequencing will become the new standard for mutagenesis testing as it is rapid, unbiased and very accurate. Human cell lines will be the most relevant for this purpose, but the chicken DT40 line is a good choice due to its stable diploid genome and its well-studied DNA repair properties [29, 48]. Indeed, the dependence of a particular mutagenic process on various DNA repair or replicative DNA damage bypass pathways is readily testable using the wide range of available DT40 mutant cell lines, which have been used for genotoxicity screening [49].

The first outcome of using the whole genome as a mutagenesis assay was the determination of the spontaneous mutation rate at 2.3 × 10^–10^ mutations per base per cell division, the first such measurement in a vertebrate cell line. Remarkably, this is the same order of magnitude as measurements obtained from budding yeast (3.6 × 10^–10^ [50]; 1.67 × 10^–10^ [51]) or *C. elegans* (6.7 × 10^–10^ [8]). The mutation rate in the human paternal germline is about two SNVs per year, while an average of 14.2 de novo mutations arise in the maternal germline in total [52]. Using estimates that cell divisions take place every 15–16 days in the human paternal germline, and there are a total of 22–23 divisions in the maternal germline [53], the mean mutation rates can be estimated as 0.17 × 10^–10^ and 1.1 × 10^–10^ per base pair per cell division in the human paternal and maternal germlines, respectively. The lack of dependence on maternal age [52] suggests that spontaneous mutations mostly arise during cell proliferation, and the similarity of these mutation rates throughout eukaryotes may be due to constraints of cellular metabolism and the mechanism of eukaryotic DNA replication.

The mutagenic effect of cisplatin has been extensively studied in prokaryotic and eukaryotic systems, as well as in vitro [7]. Studies ranging from the replication of a defined lesion in a shuttle vector in mammalian cells [38] to whole genome sequencing of cisplatin-treated *C. elegans* worms [8] identified CC > AC base substitutions as the most common cisplatin-induced mutation. In this study, we mapped a greater number of mutations than earlier investigations, presenting a fine resolution analysis of cisplatin-induced mutations. A detailed inspection of base substitutions and short indels revealed that the vast majority of such mutations are generated at intrastrand crosslinks, the most common cisplatin DNA lesions. We showed that mutations can arise at either nucleotide of the intrastrand crosslinks as well as at the previous upstream and the next downstream position. Interstrand crosslinks, which may be more significant for the cytotoxic effect of cisplatin, had no detectable mutagenic effect.

Cyclophosphamide induced a markedly different SNV mutation spectrum than cisplatin, with the elevation primarily of T > A and C > T mutation numbers. It is challenging to explain the mutation spectrum based on the available evidence of cyclophosphamide-induced lesions [6]. C > T mutations, which show strand bias in genes, may arise from N7-guanine adducts of the cyclophosphamide metabolite phosphoramide mustard [54]. However, while the N7-guanine adducts of cisplatin typically lead to C > A changes opposite the lesion, the C > T mutations caused by cyclophosphamide suggest a different mutagenic mechanism. As no adducts have been detected on adenine or thymine bases, the T > A mutations, which have also been observed in lacI reporter genes of cyclophosphamide-treated mice [55], may instead be caused by the common cyclophosphamide-induced phosphotriester adducts on the DNA backbone [56]. Indeed, phosphotriester adducts show some base preference for neighbouring thymines, and pyrimidine bases in general [57]. Phosphotriester adducts are very inefficiently repaired, which could explain the lack of strand bias of T > A mutations.

Single-strand adducts are repaired primarily by base excision repair and nucleotide excision repair. Both mechanisms are expected to mostly produce an error-free outcome. Unless the lesions miscode directly, the main cause of mutagenesis is DNA replication that uses the damaged strand as template, termed translesion synthesis (TLS). This is typically performed by specialised translesion polymerases; indeed, while the replicative polymerases δ or ε cannot bypass a GG cisplatin adduct [58], polymerase η and ζ together can bypass this lesion with a classical two-polymerase mechanism [59]. Our large dataset of cisplatin-induced mutations shows that mutations on the newly synthesised strand can appear in the position immediately upstream of the lesion as well as opposite the lesion, for example we observed NCC > ACC (GGN > GGT) mutations. Similarly, one-base insertions mostly appeared in the nascent strand upstream of the lesion, such as the common ACC > AACC (GGT > GGTT) insertions. The latter also suggests a mutagenic mechanism: the template base before the crosslinked adduct may not fit perfectly into the active site of the replicative polymerase, which could lead to the base pairing of the incoming nucleotide with the previous template base, causing a templated insertion as seen here (Fig. 4e). Because all the cisplatin-induced indels are 1–2 base pairs only, and are generally located at putative lesions, it is likely that they are mostly caused by translesion synthesis rather than the repair of DNA breaks or other mechanisms. TLS across cyclophosphamide lesions, which are mostly monoadducts, may be able to avoid similar template slippage, explaining the lack of indels induced by this treatment.

A significant finding of this study is that five of the investigated cytotoxic drugs were not mutagenic under the experimental conditions. Positive results were reported for these drugs in the Ames test or the *HPRT* assay [15, 17–19, 22, 24], which suggests that these assays may overamplify the mutagenic signal. A limitation of interpreting the relevance of our finding for clinical use is that data are only available for plasma concentrations during clinical treatment, which are different from the concentration reaching the cancer cell. Still, cisplatin, 5-fluorouracil and etoposide were used at levels very near their measured plasma concentrations (Table 1). Cyclophosphamide was used at a much higher concentration, presumably due to the limited ability of cytochrome P450 enzymes in DT40 lymphocytes to activate this prodrug. Hydroxyurea, gemcitabine, doxorubicin and paclitaxel were used well below their measured clinical peak plasma concentrations. However, at the treatment concentrations only 30–70 % of the cells survived, preventing us from using higher concentrations. Overall, the near-lethal doses used in our experiments are probably a reasonable model for the conditions experienced by somatic and tumour cells during clinical treatment.

Do these results help estimate the oncogenic potential of the selected drugs? Cancers that arise as direct consequence of a known external mutagen, such as melanoma and different lung cancer types, typically contain the highest number of genomic mutations, about ten per megabase [41, 60]. If the induced SNVs are indeed the main contributors to carcinogenesis, we can conclude that a similar density of largely randomly spaced mutations is required for a tumour to develop somewhere in the body, in which case the mean density of the mutagen-induced somatic mutations in all affected cells is probably lower. Precise clinical data on the number of cytotoxic treatment-induced mutations are not available, while after a four-cycle treatment regimen, cisplatin induced an average of 0.8 mutations per megabase in DT40 cells. Thus the mutagenic consequences of such treatment are comparable to those of carcinogenic environmental mutagens. The density of mutations induced by cisplatin treatment even surpassed the median mutation density of many common cancer types including breast, pancreatic and prostate cancer, AML and chronic lymphocytic leukaemia [60], suggesting that cisplatin treatment can make a major contribution to the development of secondary malignancies.

A recent report attributed CC > CA mutations to the mutagenic effect of cisplatin treatment in whole exome sequence data of cisplatin-resistant squamous cell carcinoma of the head and neck [61]. This mutation pattern does not agree with the predominance of CC > AC cisplatin mutations demonstrated by our study and an earlier report [8]. For a treatment-derived mutation pattern to be observable, significant clonal expansion must happen between the treatment and the sampling, which is less likely if the original tumours were resistant to the treatment. Consequently, we suspect that the observed CC > CA changes appeared due to a sample preparation artefact, as has been reported previously [62]. Treatment-derived mutations will be easier to detect or validate when mutation spectra from controlled experiments are available. Following in the footsteps of initial whole genome mutagenesis studies using *E. coli*, *S. cerevisiae* and *C. elegans* [8, 63, 64], our study is the first to use whole genome sequencing of vertebrate cell clones to provide clinically relevant data on the mutagenicity of pharmaceutical agents.

Treatment-induced mutations will contribute to the further evolution of the tumour and could be relevant for the evolution of resistance. The finding of newly defined cisplatin-specific mutation types as causes of the reversion of *BRCA2* mutations supports this notion, accompanied by our estimate that one genome among as few as a million cells surviving the treatment could contain any particular specific mutation. Treatments could not only cause the reactivation of mutated genes, but any other genetic change advantageous for tumour growth or treatment resistance.

## Conclusions

This study demonstrated the utility of whole genome sequencing in cell lines as a mutagenesis assay. We determined the spontaneous mutation rate in a cultured vertebrate cell line, and found it as low as the mutation rate of a range of organisms. We measured the mutagenic effect and defined the mutation spectrum caused by eight common cytotoxic agents. Our results suggest that cytotoxic treatment with the mutagenic cisplatin or cyclophosphamide can make a major contribution to the development of secondary malignancies and also directly contribute to the development of resistance. The definition of the precise mutagenic signature of these drugs will help assaying their mutagenic effect in post-treatment tumour samples to provide further information. Based on the lack of a detectable increase in genomic mutations following treatment with hydroxyurea, gemcitabine, 5-fluorouracil, doxorubicin or paclitaxel, it is less likely that base substitution and small insertion/deletion mutations caused by these drugs make a significant contribution to tumorigenesis. Further confirmation of these results and the expansion of mutagenesis studies to other cancer therapeutics could influence the choice of curative cancer treatment regimens, particularly in childhood cancer.

## Methods

### Cell culture and drug treatments

The wild type DT40 cell line used in this study was obtained from the laboratory of Dr Julian E. Sale, MRC Laboratory of Molecular Biology, Cambridge, UK, and its complete genome sequence has been published [48]. Cells were grown at 37 °C under 5 % CO_2_ in RPMI-1640 medium supplemented with 7 % fetal bovine serum, 3 % chicken serum, 50 μM 2-mercaptoethanol and penicillin/streptomycin. Drug sensitivities were measured using colony survival assays; treated cells were plated in medium containing 1 % methylcellulose using a tenfold dilution series and surviving colonies were counted 10 days later.

For the mutagenesis experiments, four rounds of drug treatments were performed in weekly intervals. One million cells were treated each time. The chemicals were obtained from Sigma. Etoposide, 5-fluorouracil and paclitaxel were diluted from stock solutions in DMSO. The remaining drugs were dissolved in water. Cyclophosphamide and 5-fluorouracil were dissolved freshly each time before treatments. Mock-treated cells were handled in parallel without the addition of any drug. Single-cell clones were isolated by limiting dilution and grown prior to sample preparation. Six clones were selected from 96-well plates at random. Genomic DNA was prepared using the Gentra Puregene Cell Kit (Qiagen) and three of the preps were sequenced. Cisplatin sensitivity measurements were performed on four clones which included the three sequenced clones.

### Whole genome sequencing and mutation detection

Library preparation was done using either the TruSeq DNA Nano Library Preparation Kit (Illumina) or the NEBNext Ultra DNA Library Prep Kit for Illumina (New England Biolabs). Seven library pools (the starting clone and one each of mock, cisplatin, hydroxyurea, gemcitabine, etoposide and paclitaxel treated clones) were loaded on Illumina HiSeq 2500 Rapid Run flow cells (v1) and sequenced in a 2 × 150-bp paired end (PE150) format using Rapid SBS reagents. The remaining 22 samples were loaded on Illumina HiSeq 2500 v4 High Output flow cells and sequenced in a 2 × 125-bp paired end format using HiSeq SBS v4 reagents. Library preparation and DNA sequencing were done at the Research Technology Support Facility of Michigan State University, USA and at Novogene, Beijing, China.

The reads were aligned to the chicken (Gallus gallus) reference sequence Galgal4.73, using a method already described [48]. Duplicate reads were removed using the samblaster program [65]. Additionally, the aligned reads were realigned by the GATK IndelRealigner [66].

SNVs and indels were identified using the IsoMut method developed for multiple isogenic samples, using a downloadable tool [34]. Briefly, a pileup of all samples by genomic position was produced using the samtools mpileup command and a base quality filter of 30 was used to reduce sequencing noise. Data from 120 different sequenced DT40 clones were used at this step, which included the 29 samples presented in this article. To identify SNVs and indels, the pileup data were filtered at each genomic position by minimum mutated allele frequency (0.33), minimum coverage of the mutated sample [10] and minimum reference allele frequency of each other sample (0.9). These parameters were determined in an optimisation procedure using a test set [33]. The test set was obtained by comparing two sets of whole genome sequences from DT40 clones of two different genotypes, and the parameter optimisation resulted in the identification of 95 % of the test set (true-positive rate). A detailed description is given in Additional file 3. Structural variations were detected using the CREST algorithm [67].

### Mutation analysis

Insertions and deletions in homopolymer or other repeat regions were aligned to the leftmost possible position. During the analysis of indel sequence context, before adding events with complementary sequences, identified indels on the opposite strand were realigned to the right.

Cisplatin-specific and cyclophosphamide-specific mutations localising in genes and intergenic regions according to the Ensembl Galgal4.82 genomic annotation were found using BEDTools. Per gene FPKM values were obtained by aligning the publicly available SRR913007 DT40 RNA-seq dataset (Sequence Read Archive) against the Galgal4.73 reference genome using TopHat [68] and calculating the FPKM values with Cufflinks [69].

Drug-induced, 96-triplet signatures [41] encompassing all possible SNVs, were generated by pooling samples treated with the same type of chemotherapeutic. Making no assumption on which strand the mutation took place and which base of the base pair was targeted by the drug, we arrive at a total of 96 possible substitutions, which were presented as C > A, C > G, C > T, T > A, T > C and T > G changes, shown with their immediate context of 3’ and 5’ base pair in alphabetical order. Raw triplet mutation patterns for each sequenced clone are shown in Additional file 2: Figure S6. DT40 triplet signatures were adjusted by multiplying with the ratio of triplet occurrences in the human and chicken genomes (Additional file 1: Table S5, versions GRCh38.p6 and Galgal4.73, respectively) and compared to the 30 analogous human triplet signatures on the COSMIC webpage [43]. Comparisons were made using Pearson’s correlation coefficient. DT40 signatures were also compared among themselves using the same technique. Pearson correlation and cosine similarity values are given in Additional file 1: Table S6.

### Availability of supporting data

Raw sequence data for this article have been deposited with the European Nucleotide Archive under study accession number ERP014477.

### Ethics approval

Ethics approval was not required for this study.

## Additional files

Additional file 1: Supplementary Tables. **Table S1** lists the number of SNV and indel mutations found in each individual sequenced cell clone; **Table S2** provides SNV counts for each clone in a triplet context; **Tables S3** and **S4** contain dinucleotide mutation counts and indel sequence context for each cisplatin-treated clone, respectively; **Table S5** provides the trinucleotide frequencies in the chicken and human genome; **Table S6** contains numerical data for Pearson correlation and cosine similarity between observed triplet mutation patterns and COSMIC signatures. (XLSX 97 kb) Additional file 2: Supplementary Figures. **Figure S1** shows the non-normalised mean absolute numbers of SNVs in a triplet mutation context, plus the triplet frequencies used for normalisation; **Figure S2** is a sequence context analysis of GA > GT cisplatin-induced mutations; **Figure S3** shows the wider sequence context of SNVs induced by cisplatin and cyclophosphamide; **Figure S4** shows cisplatin-induced dinucleotide deletions; **Figure S5** is an analysis of mutation clustering between samples; **Figure S6** contains triplet SNV mutation spectra for each individual sequenced clone. (PDF 3484 kb) Additional file 3: Identification of somatic mutations in the whole genome sequence files using the IsoMut application. (HTML 221 kb)
