# Supplementary material for: A comprehensive survey of the mutagenic impact of common cancer cytotoxics
Source: Genome Biol. 2016 May 9;17:99. doi: 10.1186/s13059-016-0963-7 (PMC4862131; doi:10.1186/s13059-016-0963-7)
Supplement: Additional file 2: — Supplementary Figures. Figure S1 shows the non-normalised mean absolute numbers of SNVs in a triplet mutation context, plus the triplet frequencies used for normalisation; Figure S2 is a sequence context analysis of GA > GT cisplatin-induced mutations; Figure S3 shows the wider sequence context of SNVs induced by cisplatin and cyclophosphamide; Figure S4 shows cisplatin-induced dinucleotide deletions; Figure S5 is an analysis of mutation clustering between samples; Figure S6 contains triplet SNV mutation spectra for each individual sequenced clone. (PDF 3484 kb) [file 13059_2016_963_MOESM2_ESM.pdf]

Figure S1

(A) Mock treatment, cisplatin and cyclophosphamide induced SNV mutations in the context of the neighbouring bases, not normalised to the genomic frequency of base triplets. The mean of all sequenced samples is shown.
(B) The share of each triplet type in the human and chicken genome.

A

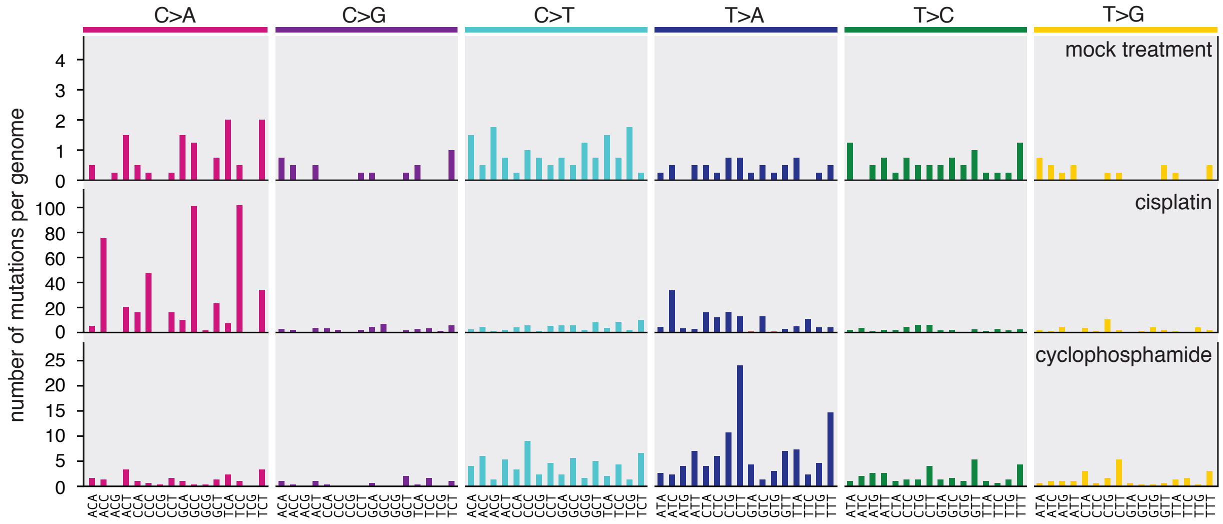

B

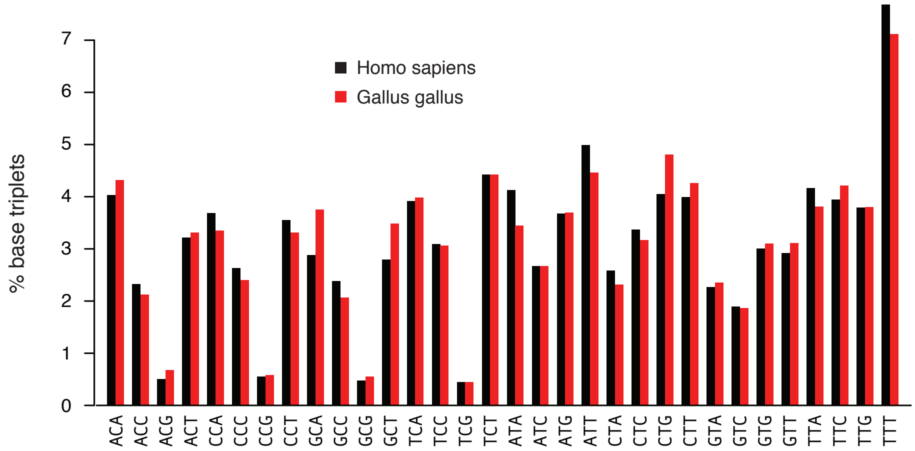

Figure S2

(A) NNGAN>NNGTN cisplatin-induced mutations separated by base context and ordered by the number of occurrence. Mutations that could be explained by GG or AG intrastrand crosslinks immediately upstream of the mutated base are shown in red, mutations at the 5' base of AG sequences are shown in blue, while mutations in the grey columns cannot be explained by putative GG or AG crosslinks overlapping or immediately upstream of the mutated position. (B-D) Schematic drawings of the replicative process that may generate GA>GT mutations in a GGA or AGA context (B); in a GAG context (C); and at GA sites with no overlapping GG or AG (D). Putative intrastrand crosslinks and monoadducts are marked; non-canonical base pairing is shown with a zig-zag symbol. The contribution of each mutation class to the total number of observed SNVs is shown, with ranges indicated where certain mutations could belong to either of two classes.

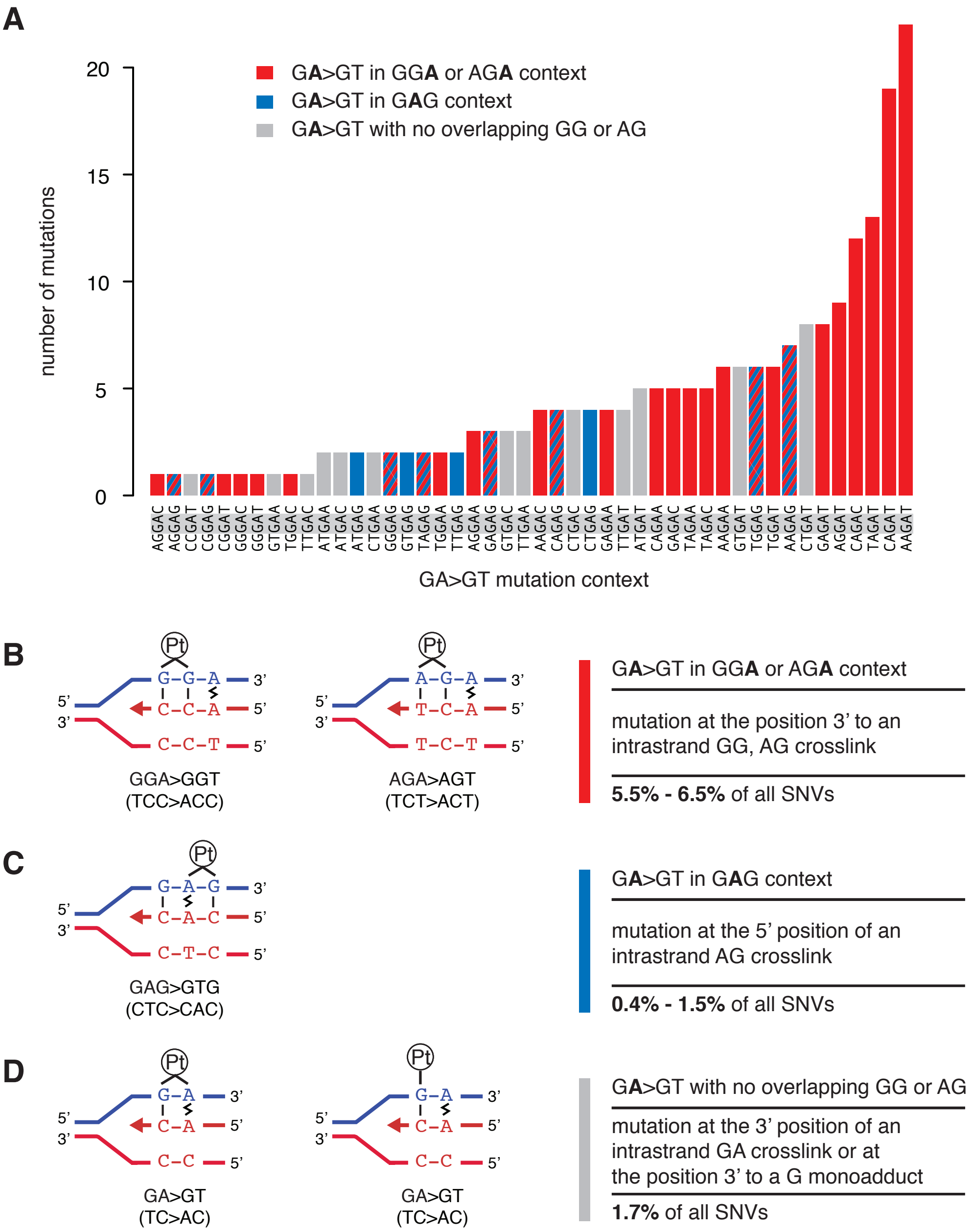

Figure S3

Sequence context of SNVs induced by cisplatin or cyclophosphamide treatment. The position of the mutated base is marked with an arrow below the panels.

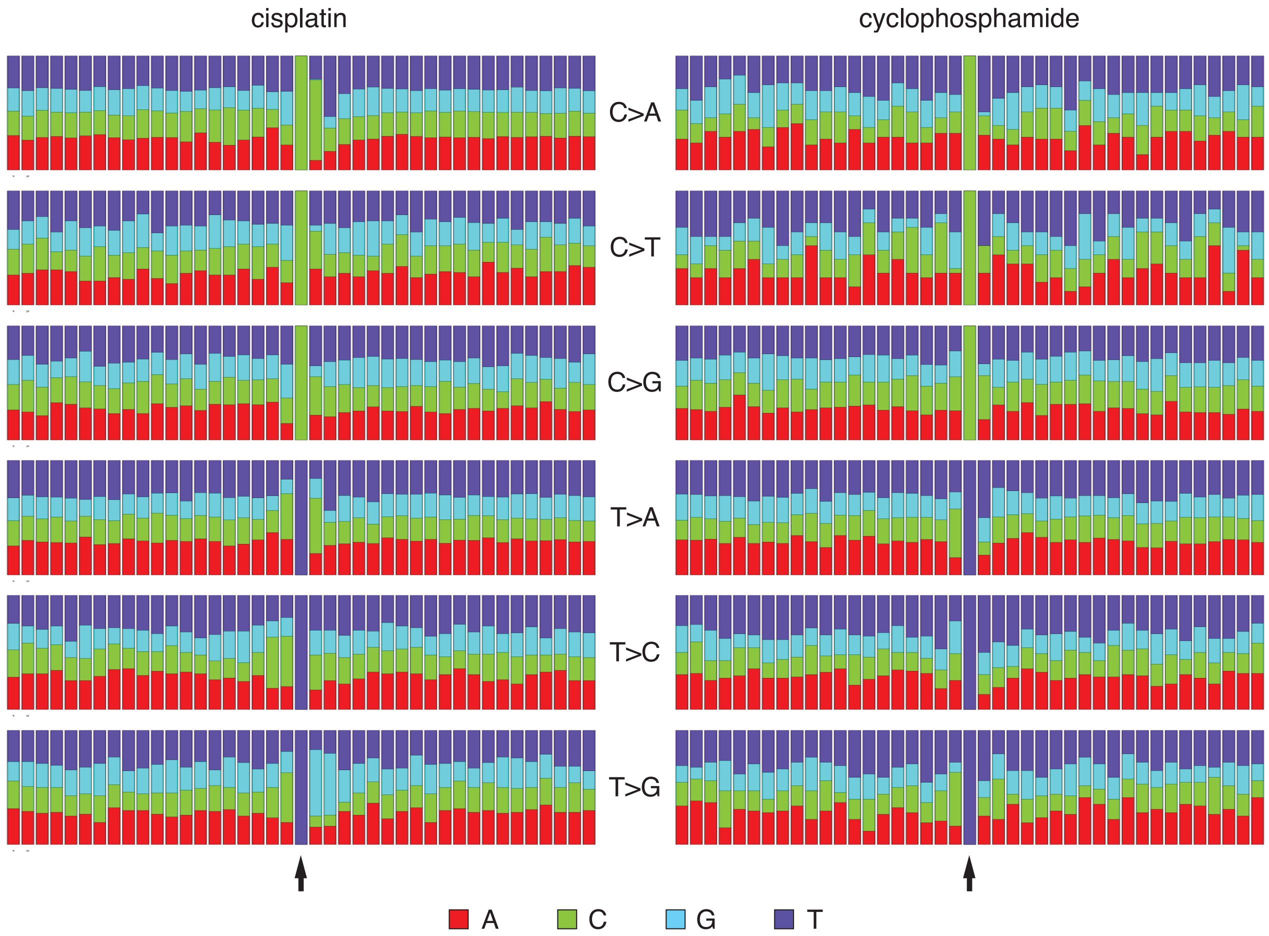

Figure S4

The number of two-base cisplatin-induced deletions, presented according to the deleted sequence, and separated per sequenced cell clone of origin.

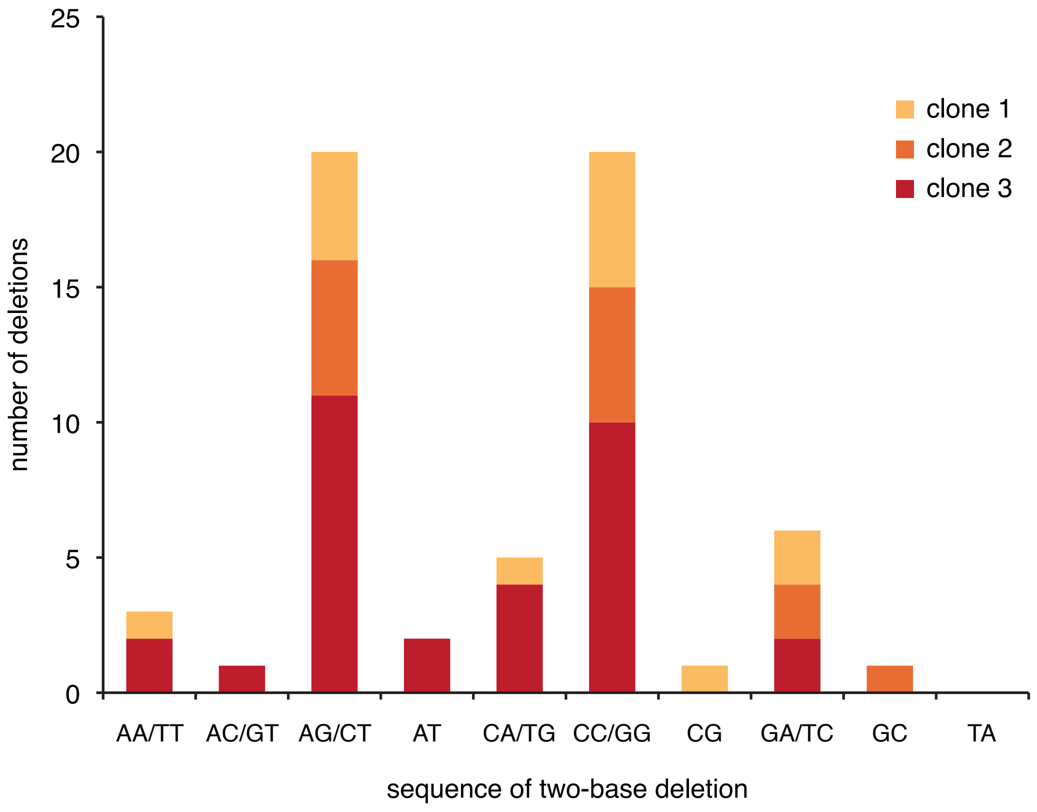

Figure S5

SNV mutations in the three samples of each treatment are overlaid on one genome, and the distance of each SNV mutation from the previous SNV on the same chromosome is plotted against the genomic position of the mutation. The colour of the dot indicates whether the previous mutation was in the same sequenced clone (blue) or a different clone (red). Mutational hotspots, which would appear as closely spaced mutations in different samples (red dots) are not seen. Thin dashed lines indicate chromosome boundaries. Chromosomes are shown in numerical order, chromosome Z is shown last on the right.

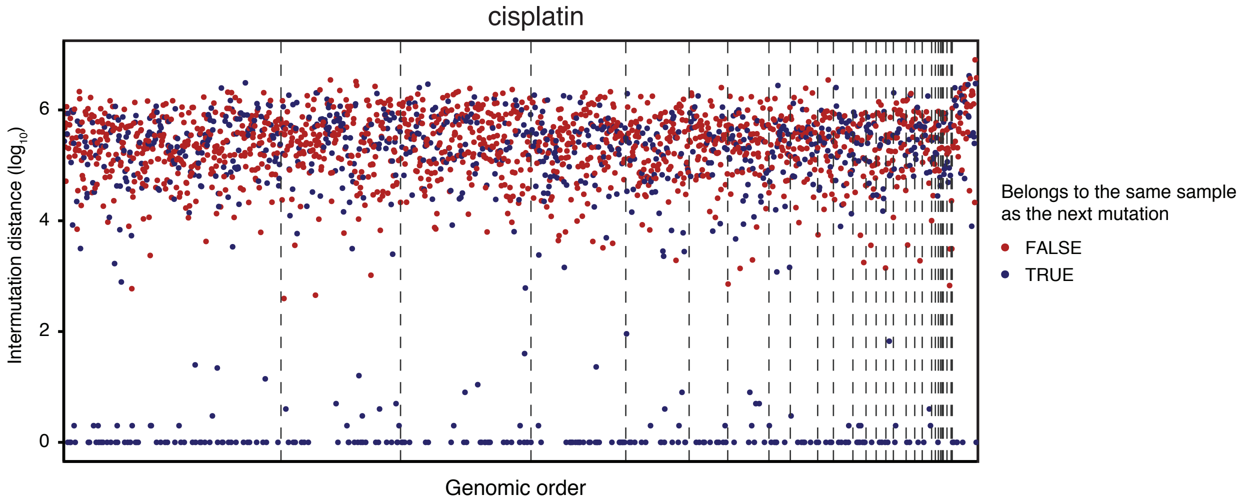

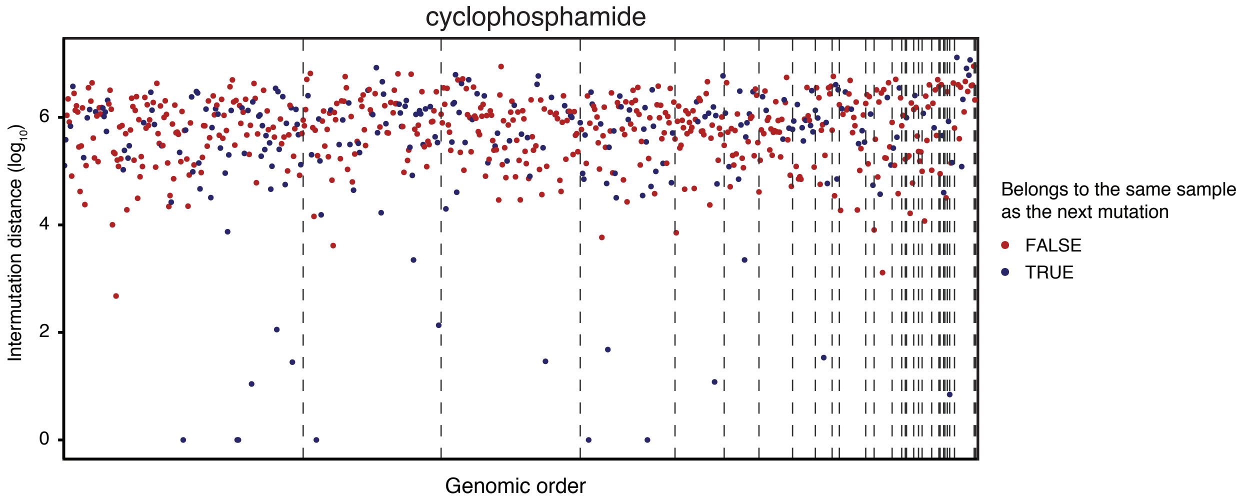

Figure S6

The number and spectrum of SNV mutations in each individual sequenced genome in the context of the neighbouring bases, not normalised to the genomic frequency of base triplets. The treatments and clone numbers are shown on the left.

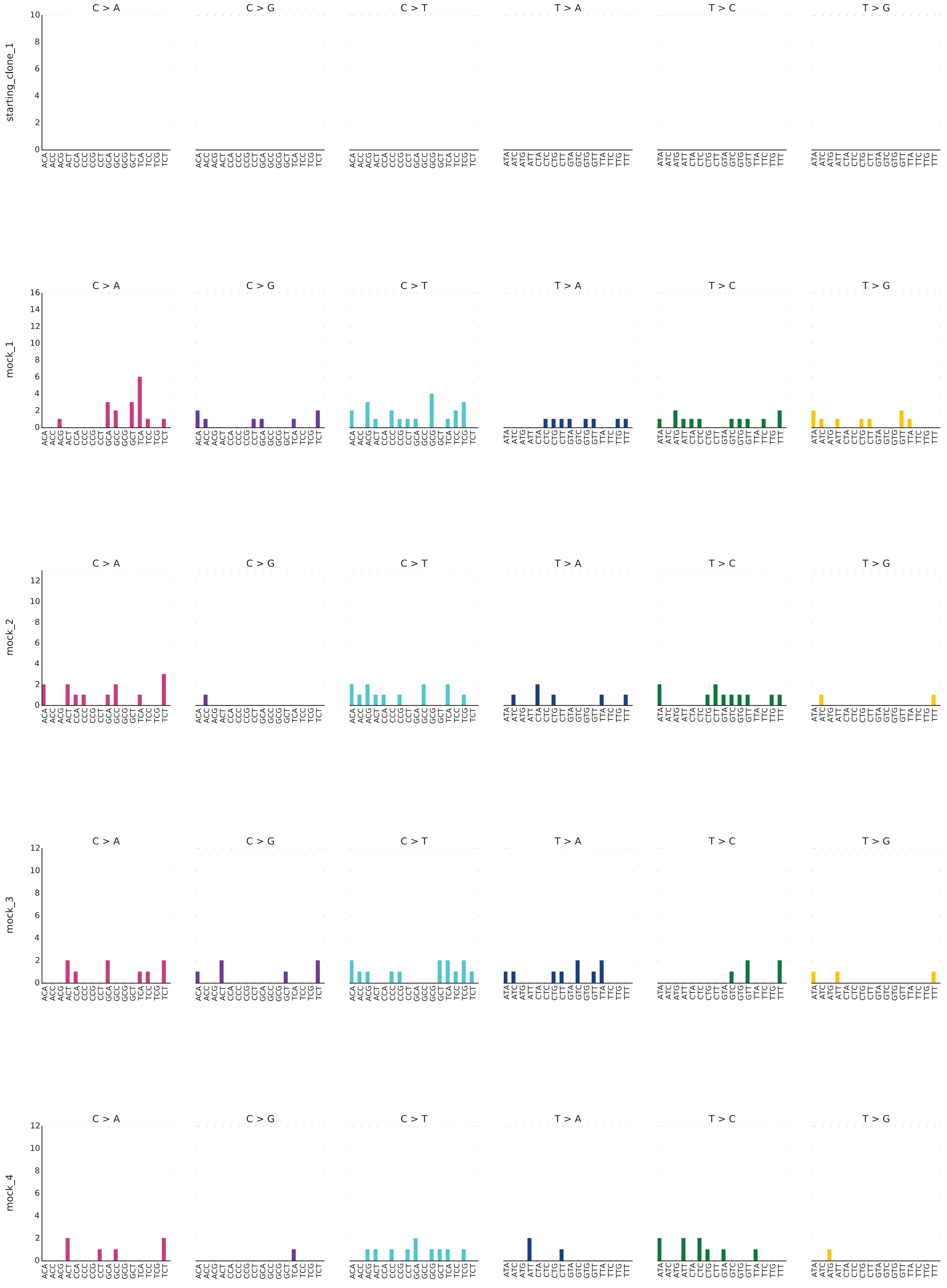

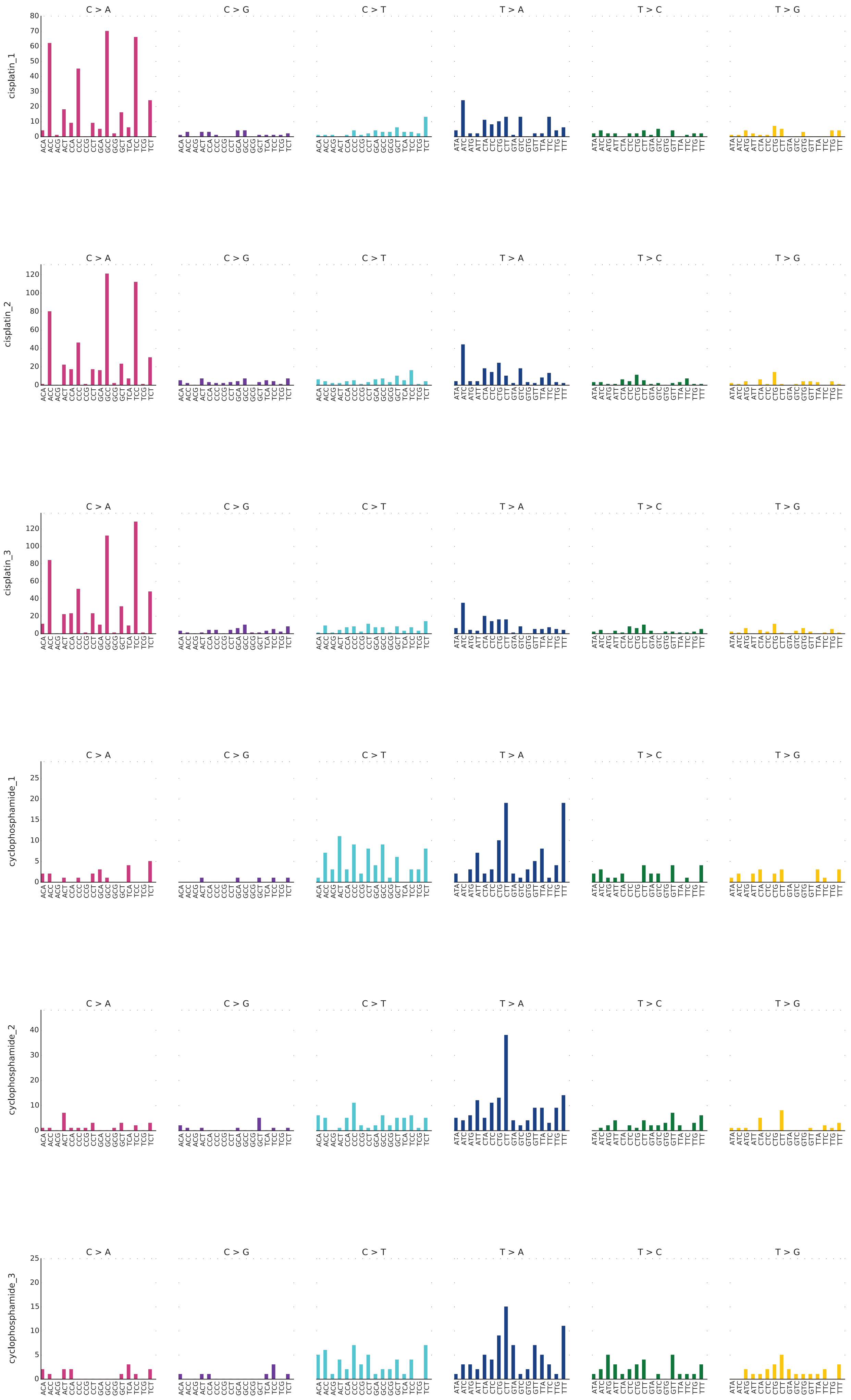
C > A
C > G
C > T
T > A
T > C
T > G
cisplatin_1
cisplatin_2
cisplatin_3
cyclophosphamide_1
cyclophosphamide_2
cyclophosphamide_3

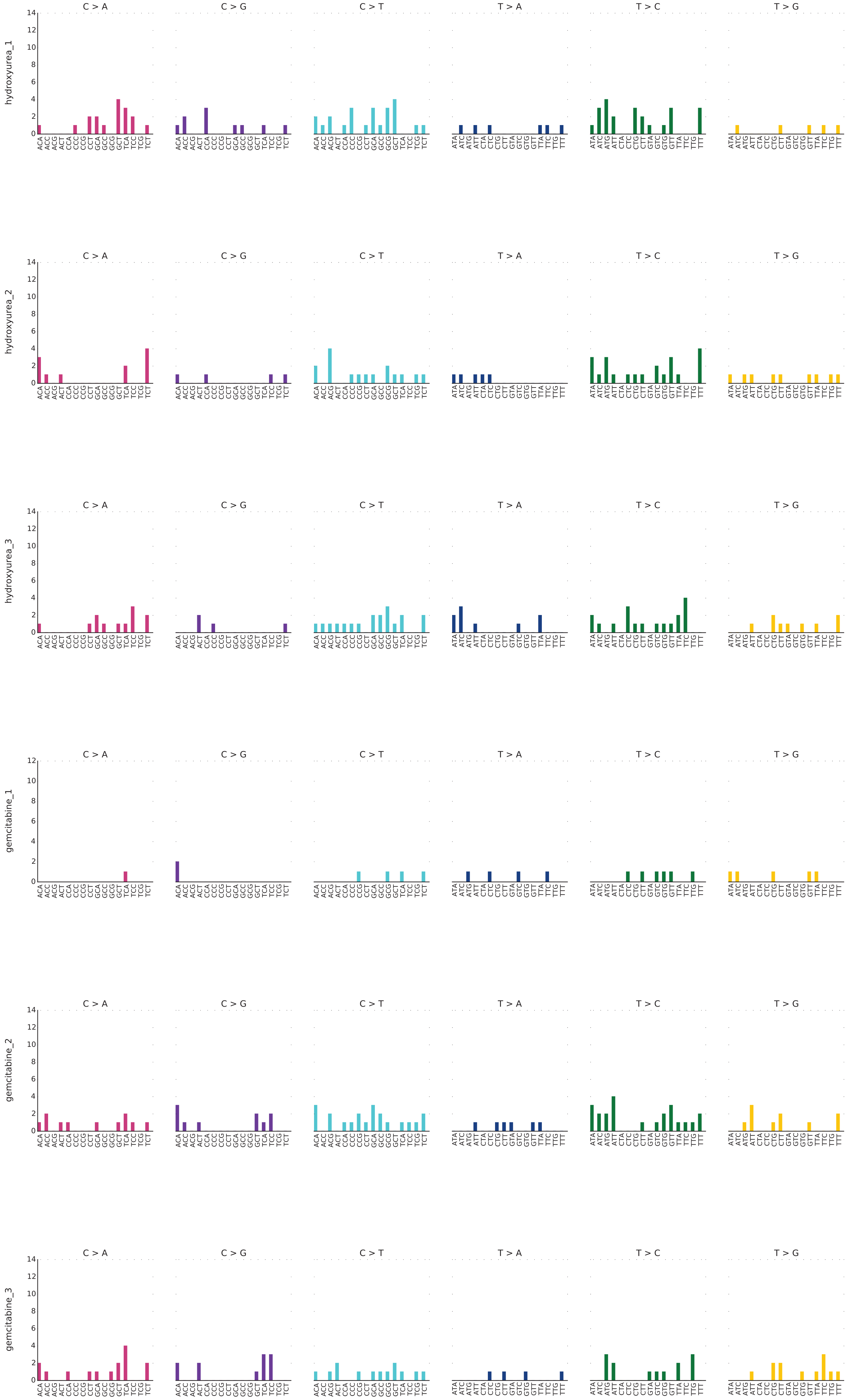
hydroxyurea_1
hydroxyurea_2
hydroxyurea_3
gemcitabine_1
gemcitabine_2
gemcitabine_3
C > A
C > G
C > T
T > A
T > C
T > G
0
2
4
6
8
10
12
14
ACA ACC ACG ACT CCA CCC CCG CCT GCA GCC GCG GCT TCA TCC TCG TCT
ATA ATC ATG ATT CTA CTC CTG CTT GTA GTC GTG GTT TTA TTC TTG TTT

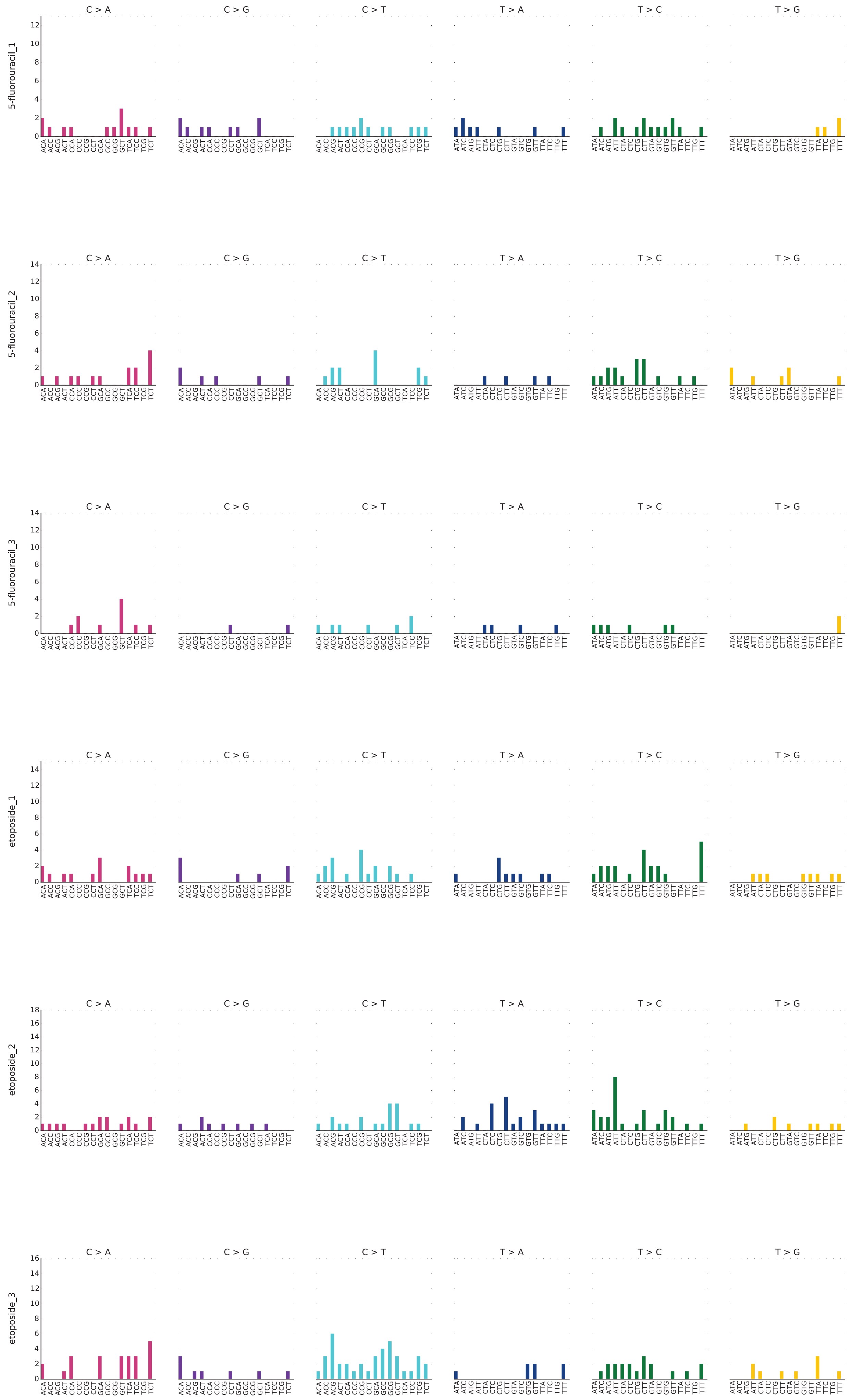
C > A
C > G
C > T
T > A
T > C
T > G
5-fluorouracil_1
5-fluorouracil_2
5-fluorouracil_3
etoposide_1
etoposide_2
etoposide_3

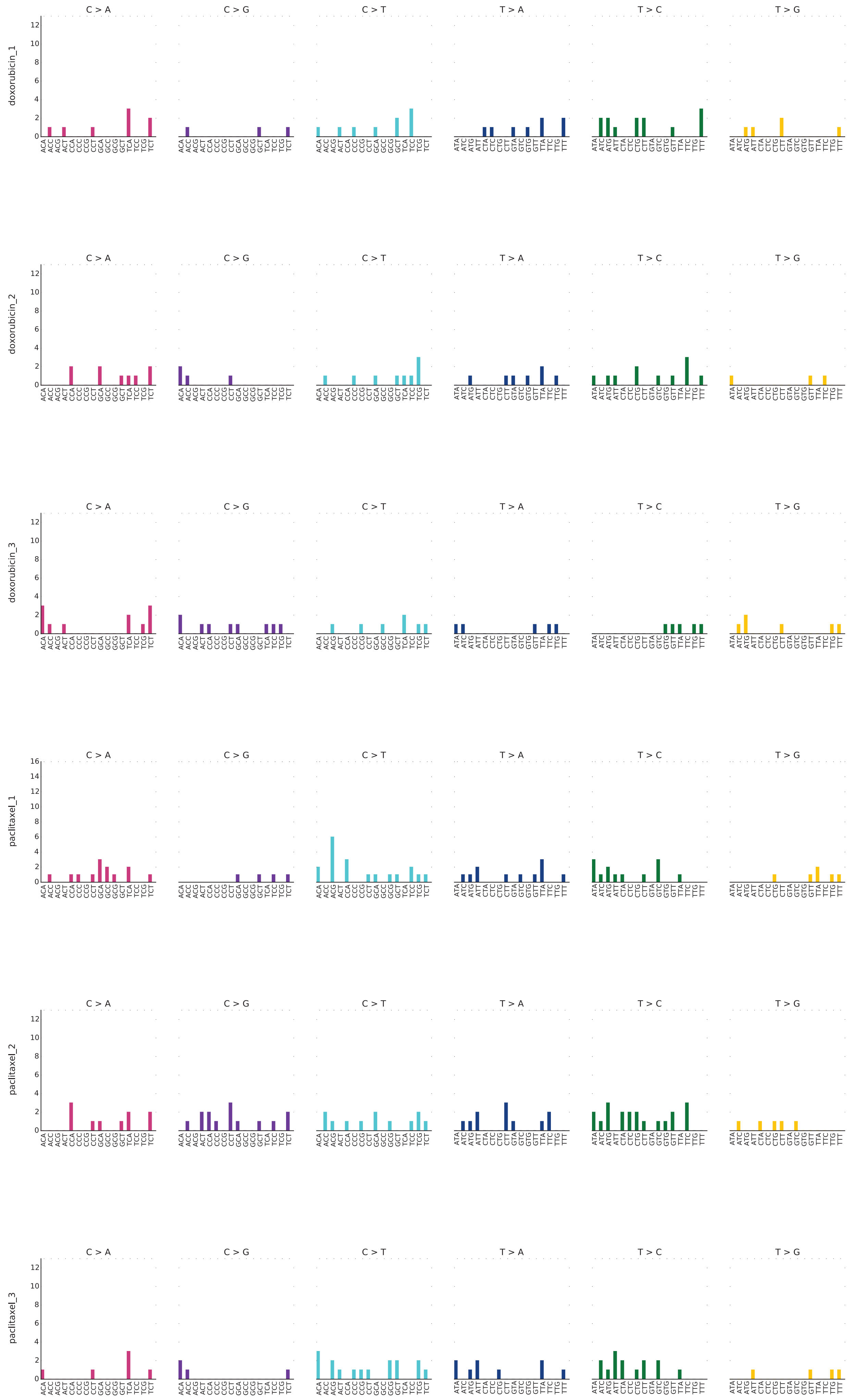
C > A
C > G
C > T
T > A
T > C
T > G
doxorubicin_1
doxorubicin_2
doxorubicin_3
paclitaxel_1
paclitaxel_2
paclitaxel_3
