# Supplementary material for: A comprehensive survey of the mutagenic impact of common cancer cytotoxics
Source: Genome Biol. 2016 May 9;17:99. doi: 10.1186/s13059-016-0963-7 (PMC4862131; doi:10.1186/s13059-016-0963-7)
Supplement: Additional file 3: — Identification of somatic mutations in the whole genome sequence files using the IsoMut application. (HTML 221 kb) [file 13059_2016_963_MOESM3_ESM.html]

IsoMut\_mutation\_detection\_pipeline


# Additional file 3 - Somatic mutation detection in a group of isogenic samples using IsoMut¶

## TITLE¶

### *A Comprehensive Survey of the Mutagenic Impact of Common Cancer Cytotoxics*¶

## AUTHORS¶

### *Bernadett Szikriszt, Ádám Póti, Orsolya Pipek, Marcin Krzystanek, Nnennaya Kanu, János Molnár, Dezső Ribli, Zoltán Szeltner, Gábor E. Tusnády, István Csabai, Zoltán Szállási, Charles Swanton and Dávid Szüts*¶

---


---

### Dataset:¶

- SNV and indel detection was carried out on a group of isogenic samples consisting of 120 differently treated and starting clone samples.

### Filtering parameters:¶

- Filtering parameters used by IsoMut were determined by carrying out a simple optimization process, the details of which can be found below.

### Usage of IsoMut:¶

- The IsoMut tool was obtained by downloading it from its github repository.
- Compiling was done using the gcc command.
- The *isomut\_example\_script.py* file was modified to fit our current dataset:

  - path names and sample names were changed
  - some filtering parameters were changed from their default values:
    - min\_sample\_freq = 0.33
    - min\_other\_ref\_freq = 0.90
    - cov\_limit = 10

---


---

### Importing python modules¶

In [11]:

```
import os
```

### Optimizing filtering parameters¶

To determine a suitable parameter set for running IsoMut a simple optimization process was carried out using two reference test sets of validated mutations (details of which can be found in (Pipek et al., manuscript in preparation)) and control samples of starting clones and identical samples in which no unique mutations are expected to be found. For different filtering *p* parameter sets, seven different *q* quality values were determined, two of which describe the number of missed validated mutations from the reference test sets (‘false negatives’) while the other five accounting for the number of mutations found in starting clones and identical samples (‘false positives’). The parameter set that yielded the lowest total sum of falsely identified mutations (p[93]) was chosen for the purposes of this paper.

The tested *p* parameter sets and the resulting *q* values can be found below with the total sum of falsely identified mutations.

In [2]:

```

```

```
p[0] = [ 0 0.2 0.75 ]	q[0] = [ 18 1560 0 3285 28359 2146 4971 ]	sum(q) = 40339
p[1] = [ 0 0.2 0.8 ]	q[1] = [ 20 1101 0 2575 26883 1712 4377 ]	sum(q) = 36668
p[2] = [ 0 0.2 0.85 ]	q[2] = [ 21 873 2 2127 25125 1464 3877 ]	sum(q) = 33489
p[3] = [ 0 0.2 0.9 ]	q[3] = [ 31 751 5 1874 22447 1302 3407 ]	sum(q) = 29817
p[4] = [ 0 0.2 0.95 ]	q[4] = [ 215 619 22 1572 16009 1055 2610 ]	sum(q) = 22102
p[5] = [ 0 0.25 0.75 ]	q[5] = [ 26 606 1 1360 11055 833 1880 ]	sum(q) = 15761
p[6] = [ 0 0.25 0.8 ]	q[6] = [ 28 448 1 1106 10477 697 1678 ]	sum(q) = 14435
p[7] = [ 0 0.25 0.85 ]	q[7] = [ 29 375 3 941 9753 607 1471 ]	sum(q) = 13179
p[8] = [ 0 0.25 0.9 ]	q[8] = [ 39 333 6 835 8594 538 1285 ]	sum(q) = 11630
p[9] = [ 0 0.25 0.95 ]	q[9] = [ 222 285 23 714 6366 436 1014 ]	sum(q) = 9060
p[10] = [ 0 0.3 0.75 ]	q[10] = [ 52 204 1 492 3602 312 590 ]	sum(q) = 5253
p[11] = [ 0 0.3 0.8 ]	q[11] = [ 54 155 1 404 3391 262 519 ]	sum(q) = 4786
p[12] = [ 0 0.3 0.85 ]	q[12] = [ 55 128 3 356 3111 228 439 ]	sum(q) = 4320
p[13] = [ 0 0.3 0.9 ]	q[13] = [ 63 114 6 321 2701 200 381 ]	sum(q) = 3786
p[14] = [ 0 0.3 0.95 ]	q[14] = [ 238 103 23 277 2066 161 301 ]	sum(q) = 3169
p[15] = [ 0 0.33 0.75 ]	q[15] = [ 71 182 2 457 3536 296 563 ]	sum(q) = 5107
p[16] = [ 0 0.33 0.8 ]	q[16] = [ 73 148 2 395 3355 256 511 ]	sum(q) = 4740
p[17] = [ 0 0.33 0.85 ]	q[17] = [ 74 126 4 354 3088 227 438 ]	sum(q) = 4311
p[18] = [ 0 0.33 0.9 ]	q[18] = [ 82 112 7 321 2682 200 381 ]	sum(q) = 3785
p[19] = [ 0 0.33 0.95 ]	q[19] = [ 253 101 24 277 2053 161 301 ]	sum(q) = 3170
p[20] = [ 0 0.35 0.75 ]	q[20] = [ 95 60 3 181 980 78 147 ]	sum(q) = 1544
p[21] = [ 0 0.35 0.8 ]	q[21] = [ 96 42 3 140 911 57 128 ]	sum(q) = 1377
p[22] = [ 0 0.35 0.85 ]	q[22] = [ 97 32 5 118 826 45 104 ]	sum(q) = 1227
p[23] = [ 0 0.35 0.9 ]	q[23] = [ 105 29 8 103 706 38 92 ]	sum(q) = 1081
p[24] = [ 0 0.35 0.95 ]	q[24] = [ 273 27 25 93 530 32 78 ]	sum(q) = 1058
p[25] = [ 5 0.2 0.75 ]	q[25] = [ 18 1151 1 2303 19047 1464 3423 ]	sum(q) = 27407
p[26] = [ 5 0.2 0.8 ]	q[26] = [ 20 726 1 1628 17824 1064 2886 ]	sum(q) = 24149
p[27] = [ 5 0.2 0.85 ]	q[27] = [ 21 530 3 1235 16593 873 2518 ]	sum(q) = 21773
p[28] = [ 5 0.2 0.9 ]	q[28] = [ 31 441 6 1053 14912 772 2202 ]	sum(q) = 19417
p[29] = [ 5 0.2 0.95 ]	q[29] = [ 215 350 22 863 10266 623 1640 ]	sum(q) = 13979
p[30] = [ 5 0.25 0.75 ]	q[30] = [ 26 197 2 378 1743 151 332 ]	sum(q) = 2829
p[31] = [ 5 0.25 0.8 ]	q[31] = [ 28 73 2 159 1418 49 187 ]	sum(q) = 1916
p[32] = [ 5 0.25 0.85 ]	q[32] = [ 29 32 4 49 1221 16 112 ]	sum(q) = 1463
p[33] = [ 5 0.25 0.9 ]	q[33] = [ 39 23 7 14 1059 8 80 ]	sum(q) = 1230
p[34] = [ 5 0.25 0.95 ]	q[34] = [ 222 16 23 5 623 4 44 ]	sum(q) = 937
p[35] = [ 5 0.3 0.75 ]	q[35] = [ 52 65 2 113 400 53 100 ]	sum(q) = 785
p[36] = [ 5 0.3 0.8 ]	q[36] = [ 54 29 2 39 295 20 48 ]	sum(q) = 487
p[37] = [ 5 0.3 0.85 ]	q[37] = [ 55 16 4 12 244 6 21 ]	sum(q) = 358
p[38] = [ 5 0.3 0.9 ]	q[38] = [ 63 14 7 4 194 2 13 ]	sum(q) = 297
p[39] = [ 5 0.3 0.95 ]	q[39] = [ 238 12 23 1 120 1 8 ]	sum(q) = 403
p[40] = [ 5 0.33 0.75 ]	q[40] = [ 71 43 3 78 334 37 73 ]	sum(q) = 639
p[41] = [ 5 0.33 0.8 ]	q[41] = [ 73 22 3 30 259 14 40 ]	sum(q) = 441
p[42] = [ 5 0.33 0.85 ]	q[42] = [ 74 14 5 10 221 5 20 ]	sum(q) = 349
p[43] = [ 5 0.33 0.9 ]	q[43] = [ 82 12 8 4 175 2 13 ]	sum(q) = 296
p[44] = [ 5 0.33 0.95 ]	q[44] = [ 253 10 24 1 107 1 8 ]	sum(q) = 404
p[45] = [ 5 0.35 0.75 ]	q[45] = [ 95 28 4 53 127 22 36 ]	sum(q) = 365
p[46] = [ 5 0.35 0.8 ]	q[46] = [ 96 15 4 18 90 7 23 ]	sum(q) = 253
p[47] = [ 5 0.35 0.85 ]	q[47] = [ 97 9 6 4 70 2 12 ]	sum(q) = 200
p[48] = [ 5 0.35 0.9 ]	q[48] = [ 105 8 9 2 53 1 6 ]	sum(q) = 184
p[49] = [ 5 0.35 0.95 ]	q[49] = [ 273 7 25 1 26 1 2 ]	sum(q) = 335
p[50] = [ 7 0.2 0.75 ]	q[50] = [ 19 627 1 1111 5783 542 1036 ]	sum(q) = 9119
p[51] = [ 7 0.2 0.8 ]	q[51] = [ 21 235 1 489 4840 187 579 ]	sum(q) = 6352
p[52] = [ 7 0.2 0.85 ]	q[52] = [ 22 94 3 172 4255 57 368 ]	sum(q) = 4971
p[53] = [ 7 0.2 0.9 ]	q[53] = [ 32 46 6 58 3831 23 269 ]	sum(q) = 4265
p[54] = [ 7 0.2 0.95 ]	q[54] = [ 216 29 22 24 2247 11 153 ]	sum(q) = 2702
p[55] = [ 7 0.25 0.75 ]	q[55] = [ 27 182 2 353 1511 143 303 ]	sum(q) = 2521
p[56] = [ 7 0.25 0.8 ]	q[56] = [ 29 65 2 145 1210 45 166 ]	sum(q) = 1662
p[57] = [ 7 0.25 0.85 ]	q[57] = [ 30 27 4 44 1034 13 99 ]	sum(q) = 1251
p[58] = [ 7 0.25 0.9 ]	q[58] = [ 40 19 7 13 907 7 71 ]	sum(q) = 1064
p[59] = [ 7 0.25 0.95 ]	q[59] = [ 223 14 23 5 528 3 37 ]	sum(q) = 833
p[60] = [ 7 0.3 0.75 ]	q[60] = [ 53 50 2 88 168 45 71 ]	sum(q) = 477
p[61] = [ 7 0.3 0.8 ]	q[61] = [ 55 21 2 25 87 16 27 ]	sum(q) = 233
p[62] = [ 7 0.3 0.85 ]	q[62] = [ 56 11 4 7 57 3 8 ]	sum(q) = 146
p[63] = [ 7 0.3 0.9 ]	q[63] = [ 64 10 7 3 42 1 4 ]	sum(q) = 131
p[64] = [ 7 0.3 0.95 ]	q[64] = [ 239 10 23 1 25 0 1 ]	sum(q) = 299
p[65] = [ 7 0.33 0.75 ]	q[65] = [ 72 28 3 53 102 29 44 ]	sum(q) = 331
p[66] = [ 7 0.33 0.8 ]	q[66] = [ 74 14 3 16 51 10 19 ]	sum(q) = 187
p[67] = [ 7 0.33 0.85 ]	q[67] = [ 75 9 5 5 34 2 7 ]	sum(q) = 137
p[68] = [ 7 0.33 0.9 ]	q[68] = [ 83 8 8 3 23 1 4 ]	sum(q) = 130
p[69] = [ 7 0.33 0.95 ]	q[69] = [ 254 8 24 1 12 0 1 ]	sum(q) = 300
p[70] = [ 7 0.35 0.75 ]	q[70] = [ 96 22 4 38 55 19 28 ]	sum(q) = 262
p[71] = [ 7 0.35 0.8 ]	q[71] = [ 97 12 4 10 28 5 15 ]	sum(q) = 171
p[72] = [ 7 0.35 0.85 ]	q[72] = [ 98 7 6 3 17 1 7 ]	sum(q) = 139
p[73] = [ 7 0.35 0.9 ]	q[73] = [ 106 6 9 2 9 0 4 ]	sum(q) = 136
p[74] = [ 7 0.35 0.95 ]	q[74] = [ 274 6 25 1 4 0 1 ]	sum(q) = 311
p[75] = [ 10 0.2 0.75 ]	q[75] = [ 25 543 4 941 3584 497 773 ]	sum(q) = 6367
p[76] = [ 10 0.2 0.8 ]	q[76] = [ 27 185 4 384 2792 160 372 ]	sum(q) = 3924
p[77] = [ 10 0.2 0.85 ]	q[77] = [ 27 68 6 120 2367 41 209 ]	sum(q) = 2838
p[78] = [ 10 0.2 0.9 ]	q[78] = [ 36 32 8 35 2125 15 145 ]	sum(q) = 2396
p[79] = [ 10 0.2 0.95 ]	q[79] = [ 219 24 23 17 1263 6 81 ]	sum(q) = 1633
p[80] = [ 10 0.25 0.75 ]	q[80] = [ 33 141 5 269 450 119 156 ]	sum(q) = 1173
p[81] = [ 10 0.25 0.8 ]	q[81] = [ 35 44 5 87 235 34 49 ]	sum(q) = 489
p[82] = [ 10 0.25 0.85 ]	q[82] = [ 35 17 7 20 152 6 15 ]	sum(q) = 252
p[83] = [ 10 0.25 0.9 ]	q[83] = [ 44 13 9 5 128 2 5 ]	sum(q) = 206
p[84] = [ 10 0.25 0.95 ]	q[84] = [ 226 12 24 2 72 1 1 ]	sum(q) = 338
p[85] = [ 10 0.3 0.75 ]	q[85] = [ 58 43 5 73 118 40 59 ]	sum(q) = 396
p[86] = [ 10 0.3 0.8 ]	q[86] = [ 60 17 5 15 56 15 22 ]	sum(q) = 190
p[87] = [ 10 0.3 0.85 ]	q[87] = [ 60 10 7 5 36 3 7 ]	sum(q) = 128
p[88] = [ 10 0.3 0.9 ]	q[88] = [ 68 10 9 2 28 1 4 ]	sum(q) = 122
p[89] = [ 10 0.3 0.95 ]	q[89] = [ 242 10 24 1 15 0 1 ]	sum(q) = 293
p[90] = [ 10 0.33 0.75 ]	q[90] = [ 77 21 6 38 52 24 32 ]	sum(q) = 250
p[91] = [ 10 0.33 0.8 ]	q[91] = [ 79 10 6 6 20 9 14 ]	sum(q) = 144
p[92] = [ 10 0.33 0.85 ]	q[92] = [ 79 8 8 3 13 2 6 ]	sum(q) = 119
p[93] = [ 10 0.33 0.9 ]	q[93] = [ 78 8 8 2 6 1 4 ]	sum(q) = 107
p[94] = [ 10 0.33 0.95 ]	q[94] = [ 253 8 24 1 2 0 1 ]	sum(q) = 289
p[95] = [ 10 0.35 0.75 ]	q[95] = [ 101 15 7 28 30 16 22 ]	sum(q) = 219
p[96] = [ 10 0.35 0.8 ]	q[96] = [ 102 8 7 5 14 5 11 ]	sum(q) = 152
p[97] = [ 10 0.35 0.85 ]	q[97] = [ 102 6 9 2 10 1 6 ]	sum(q) = 136
p[98] = [ 10 0.35 0.9 ]	q[98] = [ 110 6 11 2 6 0 4 ]	sum(q) = 139
p[99] = [ 10 0.35 0.95 ]	q[99] = [ 277 6 26 1 1 0 1 ]	sum(q) = 312
p[100] = [ 14 0.2 0.75 ]	q[100] = [ 66 436 12 693 1270 435 469 ]	sum(q) = 3381
p[101] = [ 14 0.2 0.8 ]	q[101] = [ 67 135 12 252 710 125 165 ]	sum(q) = 1466
p[102] = [ 14 0.2 0.85 ]	q[102] = [ 67 41 14 55 460 23 54 ]	sum(q) = 714
p[103] = [ 14 0.2 0.9 ]	q[103] = [ 76 21 15 13 396 7 23 ]	sum(q) = 551
p[104] = [ 14 0.2 0.95 ]	q[104] = [ 251 17 28 6 214 1 10 ]	sum(q) = 527
p[105] = [ 14 0.25 0.75 ]	q[105] = [ 71 113 13 184 212 102 98 ]	sum(q) = 793
p[106] = [ 14 0.25 0.8 ]	q[106] = [ 72 40 13 49 77 27 29 ]	sum(q) = 307
p[107] = [ 14 0.25 0.85 ]	q[107] = [ 72 16 15 10 33 6 10 ]	sum(q) = 162
p[108] = [ 14 0.25 0.9 ]	q[108] = [ 81 12 16 3 27 2 4 ]	sum(q) = 145
p[109] = [ 14 0.25 0.95 ]	q[109] = [ 255 12 29 2 9 1 1 ]	sum(q) = 309
p[110] = [ 14 0.3 0.75 ]	q[110] = [ 95 29 13 39 42 34 32 ]	sum(q) = 284
p[111] = [ 14 0.3 0.8 ]	q[111] = [ 96 17 13 3 14 12 12 ]	sum(q) = 167
p[112] = [ 14 0.3 0.85 ]	q[112] = [ 96 10 15 2 6 3 5 ]	sum(q) = 137
p[113] = [ 14 0.3 0.9 ]	q[113] = [ 104 10 16 2 5 1 4 ]	sum(q) = 142
p[114] = [ 14 0.3 0.95 ]	q[114] = [ 271 10 29 1 1 0 1 ]	sum(q) = 313
p[115] = [ 14 0.33 0.75 ]	q[115] = [ 114 15 13 25 23 21 20 ]	sum(q) = 231
p[116] = [ 14 0.33 0.8 ]	q[116] = [ 115 10 13 3 9 7 9 ]	sum(q) = 166
p[117] = [ 14 0.33 0.85 ]	q[117] = [ 115 8 15 2 6 2 5 ]	sum(q) = 153
p[118] = [ 14 0.33 0.9 ]	q[118] = [ 123 8 16 2 5 1 4 ]	sum(q) = 159
p[119] = [ 14 0.33 0.95 ]	q[119] = [ 286 8 29 1 1 0 1 ]	sum(q) = 326
p[120] = [ 14 0.35 0.75 ]	q[120] = [ 138 11 14 20 12 16 14 ]	sum(q) = 225
p[121] = [ 14 0.35 0.8 ]	q[121] = [ 138 8 14 3 6 5 8 ]	sum(q) = 182
p[122] = [ 14 0.35 0.85 ]	q[122] = [ 138 6 16 2 5 1 5 ]	sum(q) = 173
p[123] = [ 14 0.35 0.9 ]	q[123] = [ 146 6 17 2 4 0 4 ]	sum(q) = 179
p[124] = [ 14 0.35 0.95 ]	q[124] = [ 306 6 30 1 1 0 1 ]	sum(q) = 345


----------------------------------------

Lowest total sum of false mutations: 107
Best parameter setting: p[93] = [ 10 0.33 0.9 ]
```

### Downloading IsoMut¶

In [13]:

```
IsoMut_dir = 'IsoMut'
os.chdir(IsoMut_dir)
```

In [15]:

```
os.system("git clone https://github.com/riblidezso/isomut.git")
```

Out[15]:

```
0
```

### Compiling IsoMut¶

In [16]:

```
os.chdir("isomut/src")
os.system("gcc -O3 -c isomut_lib.c fisher.c  -W -Wall")
os.system("gcc -O3 -o isomut isomut.c isomut_lib.o  fisher.o -lm -W -Wall")
os.chdir("..")
```

Out[16]:

```
0
```

### Modifying and running *isomut\_example\_script.py*¶

In [22]:

```
#################################################
# importing the wrapper
#################################################
import sys,os
#add path for isomut_wrappers.py
#	if not running it from the isomut directory
#	change os.getcwd for the path to it
sys.path.append(os.getcwd()+'/src')
#load the parallel wrapper function
from isomut_wrappers import run_isomut

#add path for isomut, if its in the path comment/delete this line
#	if not running it from the isomut directory
#	change os.getcwd for the path to it
os.environ["PATH"] += os.pathsep + os.getcwd() +'/src'


#################################################
# defining administrative parameters
#################################################
#using parameter dictionary
params=dict()
#minimum number of blocks to run
# usually there will be 10-20 more blocks
params['n_min_block']=200
#number of concurrent processes to run
params['n_conc_blocks']=4
#genome
params['ref_fasta']="/home/ribli/input/index/gallus/Gallus_gallus.Galgal4.74.dna.toplevel.fa"
#input dir, output dir
params['input_dir']='/nagyvinyok/adat83/sotejedlik/orsi/bam_all_links/'
params['output_dir']='output/'
#the bam files used
params['bam_filenames']=[]
for i in xrange(1,8):
    params['bam_filenames'].append("DS" + "%03d"%i + "_RMdup_picard_realign.bam")
for i in xrange(9,12):
    params['bam_filenames'].append("DS" + "%03d"%i + "_RMdup_picard_realign.bam")
for i in xrange(14,17):
    params['bam_filenames'].append("DS" + "%03d"%i + "_RMdup_picard_realign.bam")
params['bam_filenames'].append("DS018_RMdup_picard_realign.bam")
params['bam_filenames'].append("DS026_RMdup_picard_realign.bam")
params['bam_filenames'].append("DS027_RMdup_picard_realign.bam")
for i in xrange(33,38):
    params['bam_filenames'].append("DS" + "%03d"%i + "_RMdup_picard_realign.bam")
for i in xrange(41,75):
    params['bam_filenames'].append("DS" + "%03d"%i + "_RMdup_picard_realign.bam")
for i in xrange(81,138):
    params['bam_filenames'].append("DS" + "%03d"%i + "_RMdup_picard_realign.bam")
for i in xrange(141,149):
    params['bam_filenames'].append("DS" + "%03d"%i + "_RMdup_picard_realign.bam")

#limit chromosomes (for references with many scaffolds)
params['chromosomes']=map(str,range(1,29))+ ['32','W','Z','MT']

#################################################
# defining mutation calling parameters
#################################################
params['min_sample_freq']=0.33
params['min_other_ref_freq']=0.9
params['cov_limit']=10
params['base_quality_limit']=30
params['min_gap_dist_snv']=0
params['min_gap_dist_indel']=20

#################################################
# running IsoMut
#################################################
run_isomut(params)
```

```
Defining parallel blocks ...
Done

blocks to run: 216
running: 1 2 3 4 5 6 7 8 9 10 11 12 13 14 15 16 17 18 19 20 21 22 23 24 25 26 27 28 29 30 31 32 33 34 35 36 37 38 39 40 41 42 43 44 45 46 47 48 49 50 51 52 53 54 55 56 57 58 59 60 61 62 63 64 65 66 67 68 69 70 71 72 73 74 75 76 77 78 79 80 81 82 83 84 85 86 87 88 89 90 91 92 93 94 95 96 97 98 99 100 101 102 103 104 105 106 107 108 109 110 111 112 113 114 115 116 117 118 119 120 121 122 123 124 125 126 127 128 129 130 131 132 133 134 135 136 137 138 139 140 141 142 143 144 145 146 147 148 149 150 151 152 153 154 155 156 157 158 159 160 161 162 163 164 165 166 167 168 169 170 171 172 173 174 175 176 177 178 179 180 181 182 183 184 185 186 187 188 189 190 191 192 193 194 195 196 197 198 199 200 201 202 203 204 205 206 207 208 209 210 211 212 213 214 215 216 
Done

Defining parallel blocks ...
Done

blocks to run: 216
running: 1 2 3 4 5 6 7 8 9 10 11 12 13 14 15 16 17 18 19 20 21 22 23 24 25 26 27 28 29 30 31 32 33 34 35 36 37 38 39 40 41 42 43 44 45 46 47 48 49 50 51 52 53 54 55 56 57 58 59 60 61 62 63 64 65 66 67 68 69 70 71 72 73 74 75 76 77 78 79 80 81 82 83 84 85 86 87 88 89 90 91 92 93 94 95 96 97 98 99 100 101 102 103 104 105 106 107 108 109 110 111 112 113 114 115 116 117 118 119 120 121 122 123 124 125 126 127 128 129 130 131 132 133 134 135 136 137 138 139 140 141 142 143 144 145 146 147 148 149 150 151 152 153 154 155 156 157 158 159 160 161 162 163 164 165 166 167 168 169 170 171 172 173 174 175 176 177 178 179 180 181 182 183 184 185 186 187 188 189 190 191 192 193 194 195 196 197 198 199 200 201 202 203 204 205 206 207 208 209 210 211 212 213 214 215 216 
Done


Done
```
